# EEPD1 Rescues Stressed Replication Forks and Maintains Genome Stability by Promoting End Resection and Homologous Recombination Repair

**DOI:** 10.1371/journal.pgen.1005675

**Published:** 2015-12-18

**Authors:** Yuehan Wu, Suk-Hee Lee, Elizabeth A. Williamson, Brian L. Reinert, Ju Hwan Cho, Fen Xia, Aruna Shanker Jaiswal, Gayathri Srinivasan, Bhavita Patel, Alexis Brantley, Daohong Zhou, Lijian Shao, Rupak Pathak, Martin Hauer-Jensen, Sudha Singh, Kimi Kong, Xaiohua Wu, Hyun-Suk Kim, Timothy Beissbarth, Jochen Gaedcke, Sandeep Burma, Jac A. Nickoloff, Robert A. Hromas

**Affiliations:** 1 Department of Medicine and the Cancer Center, University of Florida Health, Gainesville, Florida, United States of America; 2 Department of Biochemistry and Molecular Biology, Indiana University School of Medicine, Indianapolis, Indiana, United States of America; 3 Department of Radiation Oncology, Comprehensive Cancer Center, The Ohio State University, Columbus, Ohio, United States of America; 4 Department of Pharmaceutical Sciences, University of Arkansas for Medical Sciences, Little Rock, Arkansas, United States of America; 5 Department of Craniofacial Regeneration, College of Dental Medicine, Columbia University, New York, New York, United States of America; 6 Department of Molecular and Experimental Medicine, Scripps Research Institute, La Jolla, California, United States of America; 7 Department of Medical Statistics, and General, Visceral, and Pediatric Surgery, University Medical Center Göttingen, Göttingen, Germany; 8 Department of Radiation Oncology, University of Texas Southwestern, Dallas, Texas, United States of America; 9 Department of Environmental and Radiological Health Sciences, Colorado State University, Fort Collins, Colorado, United States of America; University of Washington School of Medicine, UNITED STATES

## Abstract

Replication fork stalling and collapse is a major source of genome instability leading to neoplastic transformation or cell death. Such stressed replication forks can be conservatively repaired and restarted using homologous recombination (HR) or non-conservatively repaired using micro-homology mediated end joining (MMEJ). HR repair of stressed forks is initiated by 5’ end resection near the fork junction, which permits 3’ single strand invasion of a homologous template for fork restart. This 5’ end resection also prevents classical non-homologous end-joining (cNHEJ), a competing pathway for DNA double-strand break (DSB) repair. Unopposed NHEJ can cause genome instability during replication stress by abnormally fusing free double strand ends that occur as unstable replication fork repair intermediates. We show here that the previously uncharacterized Exonuclease/Endonuclease/Phosphatase Domain-1 (EEPD1) protein is required for initiating repair and restart of stalled forks. EEPD1 is recruited to stalled forks, enhances 5’ DNA end resection, and promotes restart of stalled forks. Interestingly, EEPD1 directs DSB repair away from cNHEJ, and also away from MMEJ, which requires limited end resection for initiation. EEPD1 is also required for proper ATR and CHK1 phosphorylation, and formation of gamma-H2AX, RAD51 and phospho-RPA32 foci. Consistent with a direct role in stalled replication fork cleavage, EEPD1 is a 5’ overhang nuclease in an obligate complex with the end resection nuclease Exo1 and BLM. EEPD1 depletion causes nuclear and cytogenetic defects, which are made worse by replication stress. Depleting 53BP1, which slows cNHEJ, fully rescues the nuclear and cytogenetic abnormalities seen with EEPD1 depletion. These data demonstrate that genome stability during replication stress is maintained by EEPD1, which initiates HR and inhibits cNHEJ and MMEJ.

## Author Summary

The cell itself damages its own DNA throughout the cell cycle as a result of oxidative metabolism, and this damage creates barriers for replication fork progression. Thus, DNA replication is not a smooth and continuous process, but rather one of stalls and restarts. Therefore, proper replication fork restart is crucial to maintain the integrity of the cell’s genome, and preventing its own death or immortalization. To restart after stalling, the replication fork subverts a DNA repair pathway termed homologous recombination. Using any other pathway for fork repair will result in an unstable genome. How the homologous recombination repair pathway is initiated at the replication fork is not well defined. In this study we demonstrate the previously uncharacterized EEPD1 protein is a novel gatekeeper for the initiation of this fork repair pathway. EEPD1 promotes 5’ end resection, the initial step of homologous recombination, which also prevents alternative fork repair pathways that lead to unstable chromosomes. Thus, EEPD1 protects the integrity of the cell genome by promoting the safe homologous recombination fork repair pathway.

## Introduction

Maintaining genome stability depends on faithful DNA replication [1–3]. Since DNA damage from endogenous and exogenous sources creates barriers for the replication fork, replication is not a smooth, continuous process, but rather one of intermittent stress, with stops and restarts [4–6]. Replication fork reactivation after stalling at DNA damage is best characterized in *E*. *coli*, where forks are restarted by recombination-dependent or -independent pathways requiring RuvABC or the PriA/C complexes, respectively [5–7].

Eukaryotic replication fork restart is more complex and less understood, with the canonical repair pathway mediated by RAD51-dependent homologous recombination (HR) [1–3,8]. HR is best characterized for the repair of DNA double-strand breaks (DSBs). It is initiated by a litany of components mediating 5’ end resection to create 3’ single-stranded (SS) DNA, which then use BRCA2/RAD51 to create heteroduplexes with homologous sequences on sister chromatids [3,4,8–12]. After an invading strand re-initiates DNA synthesis, Holliday junctions may be resolved by either Gen1 or Mus81, with Slx4 serving as a scaffold [11–15]. End resection directs DSB repair toward HR, preventing the competing DSB repair pathway, classical non-homologous end-joining (cNHEJ) from occurring [16–19].

Similar to DSB repair, repair of stressed replication forks also requires 5’ end resection to initiate HR, but how this is regulated in fork repair and restart is less well defined [1–3,16,17]. End resection at a replication fork requires a free DNA double strand (DS) end structure to initiate 5’ exonuclease activity. This DNA DS end can be created at stressed forks in at least two ways: the fork can reverse into a chicken foot structure with a single DS DNA end [2,3,20], or a nuclease can cleave the fork, directly creating a free DS end [3,13,14,17]. If a stressed fork is not repaired in timely manner, it may convert into toxic structures that make fork restart difficult [1,13,14,19], leading to cell death or genome instability and neoplastic transformation [1,4,6].

Repair pathway choice at stalled forks is important for genome stability, because unopposed cNHEJ, as seen in malignancies with inherited deficiencies in HR proteins BRCA1 or BRCA2, results in fusion of these DNA DS ends at damaged replication forks [21–26]. These chromosomal fusions cause severe genome instability, resulting in catastrophic mitoses revealed as gross nuclear abnormalities including nuclear bridges and micronuclei [1,21,22,25,27]. The tumor suppressor p53-binding protein 1 (53BP1) promotes cNHEJ at least in part by preventing end-resection. Preventing cNHEJ by repressing 53BP1 rescues HR-deficient cells from these nuclear defects [21–23]

There is accumulating evidence that DSB pathway choice between cNHEJ and HR is mediated by 5’ end resection [16–18]. End resection appears to be a two-step process, with CtIP and Mre11 nucleases responsible for short end resection, and Dna2 and Exo1 catalyzing longer resection for HR [16,17,19,28,29]. It is thought that short end resection may lead to MMEJ and long range end resection to HR [17,19,30,31]. Although it is clear that end resection is important for regulating pathway choice at DSBs, key questions remain on how end resection is initiated at stressed forks.

In this study we identify a previously uncharacterized 5’ endonuclease, EEPD1 (endonuclease/exonuclease/phosphatase family domain-containing 1), by its up-regulation in embryonic stem cells after DNA damage. We found that EEPD1 initiates end resection, thereby enhancing HR at the expense of cNHEJ, and also of MMEJ. Consistent with an upstream role in end resection, EEPD1 depletion markedly reduces stress-induced ATR and Chk1 phosphorylation and the formation of RPA, gamma-H2Ax, and RAD51 foci, while NBS1, 53BP1, and BRCA1 foci are intact. Depletion of EEPD1 results in severe chromosomal abnormalities, made worse by replication stress. This places EEPD1 at the apex of pathway choice in repair of stressed replication forks, where it is required for maintenance of genome integrity.

## Results

### EEPD1 Promotes Cell Survival upon Replication Stress

In a survey of proteins induced by the topoisomerase IIα poison VP-16 in embryonic stem cells, we found that expression of EEPD1, an uncharacterized human protein (Uniprot Q7L989, AAH65518.1), was markedly increased. EEPD1 is a 569 aa protein with two amino terminal helix-hairpin-helix (HhH) DNA binding domains related to RuvA, a carboxy terminal DNase I-like domain that places it in the exonuclease-endonuclease-phosphatase (EEP) family, and a conserved D-D-N/D/E nuclease active site that overlaps with the HhH domain and the DNase I-like domain (S1 Fig) [32]. It is located at 7p14.2, but is not involved in any known neoplastic translocations (Catalogue of Somatic Mutations in Cancer). EEPD1 is evolutionarily conserved from some insects to humans and expressed at variable levels in a wide variety of primary human tissues and human cell lines (S1B Fig). It is more highly expressed in the testis, leukocytes, and brain, as are many other DNA DSB repair components [33,34]. EEPD1 depletion moderately altered cell cycle progression in asynchronous or synchronized cells (S2 Fig), increasing the fraction of cells in S and G2 phases in both situations.

**Fig 1:**
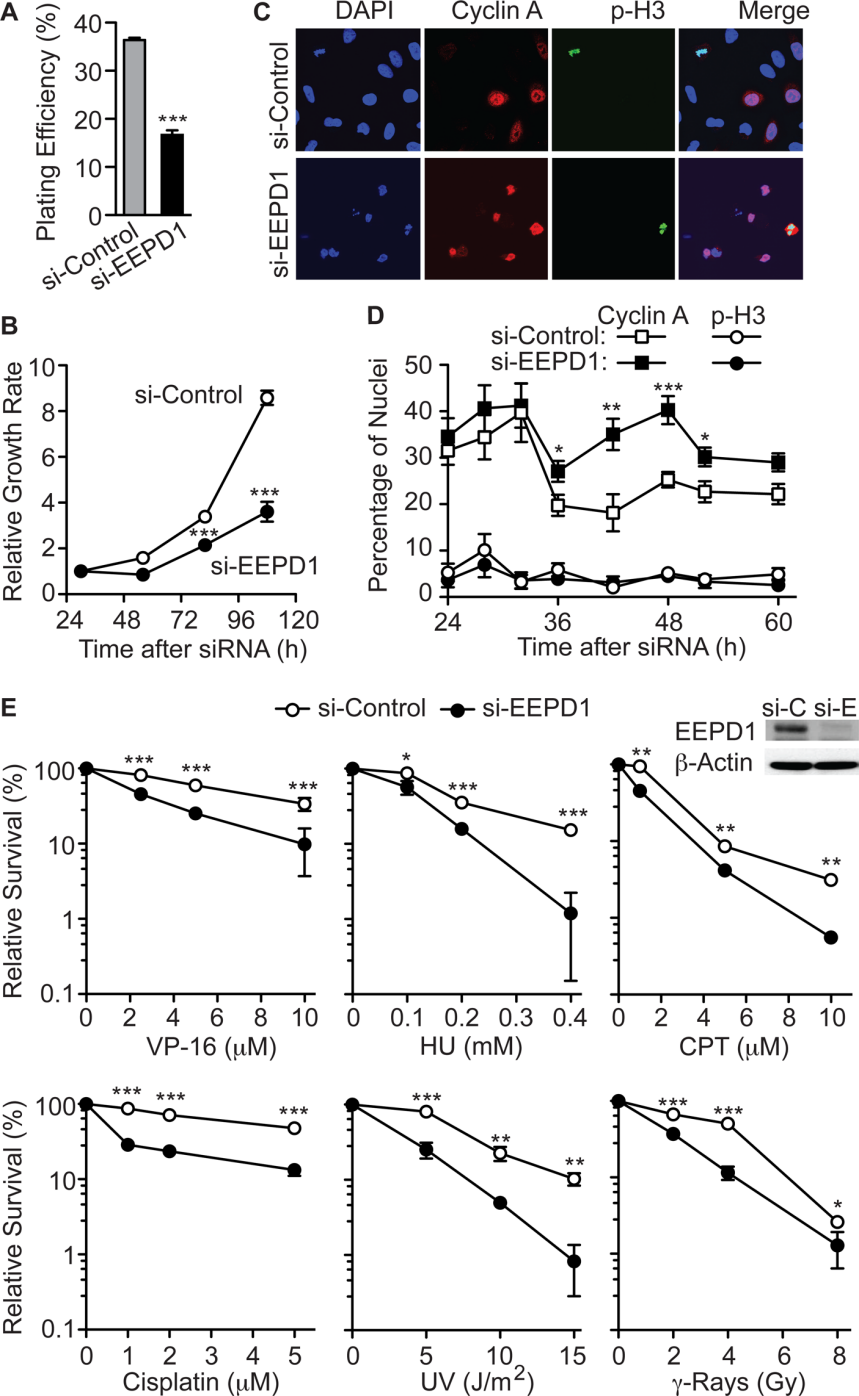
EEPD1 deficiency reduces clonogenicity and growth rate, extends S phase, and sensitizes cells to replication stress. (A) A549 cells were transfected with control siRNA or siRNA targeting EEPD1 and plating efficiencies were determined (mean ±SEM). (B) Relative growth rates of control and EEPD1 deficient cells (mean ±SEM). (C,D) Immunofluorescence microscopy of control and EEPD1 deficient A549 cells stained with DAPI, cyclin A (S phase marker), and phospho-H3 (M phase marker) at indicated times after siRNA transfection. Representative data are shown in panel C. Quantitation of 4–12 determinations (124–468 nuclei/determination) scored per time point is shown in panel D; values are mean percentages (±SEM) of cyclin A- or phospho-H3-positive nuclei. (E) Clonogenic survival of A549 cells transfected with si-EEPD1 or control siRNA, and then treated with various replication stress agents. EEPD1 repression was confirmed by Western blot, above (n = 6–9 in triplicate, means ±SEM). *, **, *** indicate P≤0.05, 0.01, 0.001 (t tests), respectively, in this and all subsequent figures unless otherwise specified.

EEPD1 alone is required for proper clonogenecity; plating efficiency is reduced by almost 50% from EEPD1 depletion alone (Fig 1A). EEPD1 deficiency also significantly slows cell growth (Fig 1B), and increases the fraction of cells expressing cyclin A, without an increase in the fraction of cells with phosphorylated histone H3 (Fig 1C and 1D). This suggested a potential role in DNA replication. To investigate whether EEPD1 is important for survival after exposure to agents that stress replication forks, we tested whether EEPD1 regulates sensitivity to VP-16, hydroxyurea (HU), camptothecin (CPT), UV light, cisplatin, and ionizing radiation (IR) (Fig 1E). EEPD1 depletion resulted in 3.5-fold less clonogenic survival after 18 h exposure to 10 uM VP-16, compared to controls. EEPD1 depletion also decreased survival to continuous 0.4 mM HU (12-fold), 18 h exposure to 10 uM CPT (6-fold), continuous 0.4 mM HU (10-fold), 18 h exposure to 10 uM CPT (6-fold), continuous 5 uM cisplatin (4-fold), 15 J/m^2^ UV (12-fold), and 4 Gy IR (4-fold).

### EEPD1 Promotes Replication Fork Restart after Replication Stress

**Fig 2:**
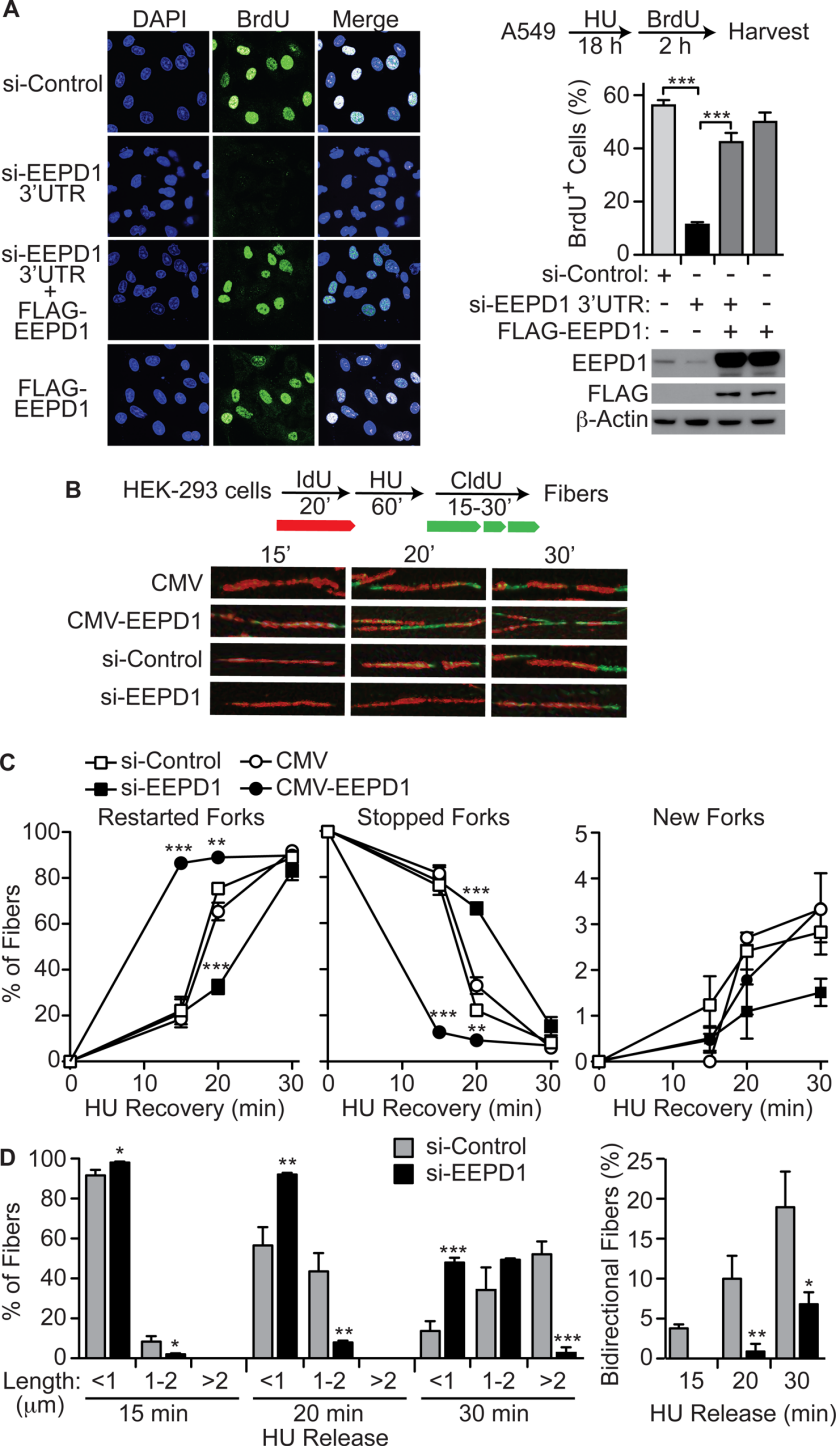
EEPD1 promotes replication fork restart after stress. (A) Replication recovery assayed as percentage of cells with ≥3 BrdU foci 2 h after release from 18 h treatment with 10 mM HU. Representative data (left) and quantitation (right) for cells transfected with control siRNA or si-EEPD1 targeted to 3’ UTR, with or without expression of siRNA-resistant FLAG-tagged EEPD1 (n = 11–23 determinations per condition, >100 cells scored/condition, means ±SEM). EEPD1 expression is shown by Western blot below for each condition. (B,C) Restart of stalled forks by DNA fiber analysis with HEK-293 cells transfected with empty vector (CMV), CMV-EEPD1 overexpression vector, control siRNA, or EEPD1 siRNA analyzed 15–30 min after release from HU replication stress. Representative images of fibers with IdU stained red and CldU stained green (B) and fiber quantitation (C) shown as percentage (means ±SD) of restarted forks (red + green fibers), stopped forks (red fibers), and new forks (green fibers) for >3 distinct determinations per condition (121–211 fibers/condition). (D) Fiber lengths and symmetry were measured in control and EEPD1 deficient cells to determine replication speed (left), and the percentage of bidirectional fibers (right), defined as red fibers with flanking green segments.

To investigate the mechanism by which EEPD1 promotes cell survival during replication stress we used two techniques to measure replication fork restart after stalling. First, BrdU incorporation into nascent DNA after release from HU replication stress was measured by immunofluorescence ([35,36]. By 2 h after release from an 18 h HU exposure, when replication fork restart was maximal in control cells (as indicated by the number of BrdU foci), EEPD1-depleted cells restarting forks were reduced by 5-fold (Fig 2A). This is a specific EEPD1 effect, as the fork restart defect in EEPD1 depleted cells was rescued by expression of an siRNA-resistant version of EEPD1 (Fig 2A).

We next used DNA fiber analysis to measure replication fork restart after release from a 1 h HU treatment, as well as replication speed and replication fork symmetry [32,35]. We found that 20 min after HU release, EEPD1 depletion reduced replication fork restart by 2.3-fold (Fig 2B and 2C). Interestingly, over-expressing EEPD1 increased fork restart; however, by 30 min nearly all forks restarted even in EEPD1-depleted cells. New fork initiation is rare under these conditions, and EEPD1 depletion had no significant effect on this endpoint (Fig 2C). By measuring fiber lengths, we determined that EEPD1 depletion significantly reduces replication speed (Fig 2D). Consistent with EEPD1 promoting fork restart, EEPD1 depletion significantly reduced the percentage of bidirectional forks, reflecting restart at both ends of a replicon (Fig 2D). These results indicate that EEPD1 accelerates restart of stressed replication forks, and that it increases the speed of replication during recovery from stress, implying that EEPD1 also assists in normal fork progression.

### EEPD1 Inhibits NHEJ and Promotes HR

**Fig 3:**
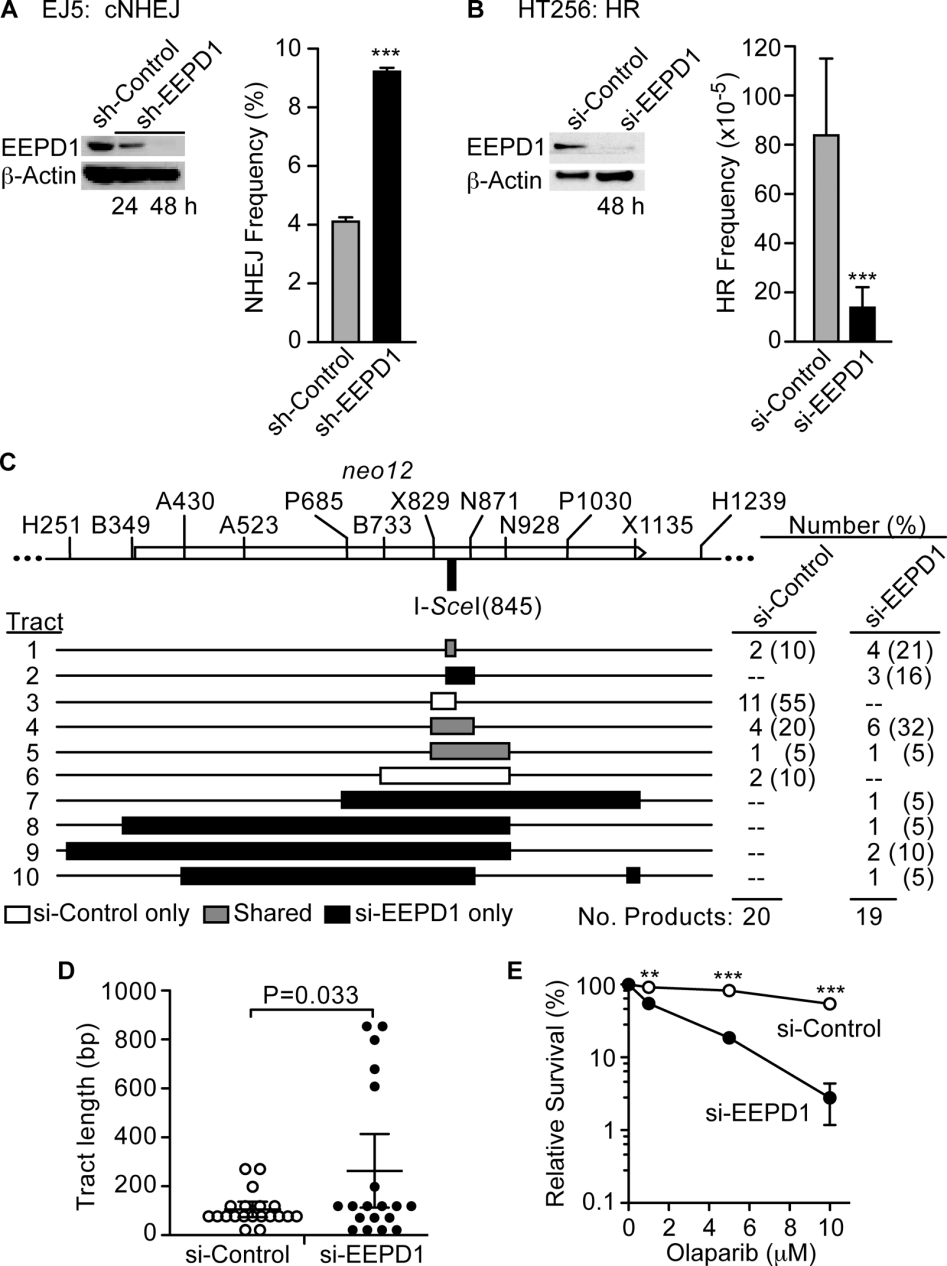
EEPD1 promotes HR DSB repair and suppresses cNHEJ. (A,B) cNHEJ repair of I-SceI-induced DSBs was measured in EJ5 cells with or without EEPD1 depletion by EEPD1 or control lentiviral shRNA, and assayed as percentages of GFP^+^ cells by flow cytometry (n = 12 in triplicate, means ±SD). HR repair of I-SceI-induced DSBs was measured in HT256 cells with or without EEPD1 depletion assayed as frequencies of G418-resistant colonies per viable cell (n = 15 in triplicate, mean ±SD). (C) HT256 gene conversion tract spectra with or without EEPD1 depletion were determined by mapping 12 silent restriction site markers in the *neo* gene. Bars indicate converted markers for each tract type; the number of products of each tract type is listed with percentages. (D) Tract lengths of each product are plotted; bars indicate mean ± 95% confidence intervals. (E) Clonogenic survival of A549 cells treated with olaparib for 18 h at indicated doses with or with EEPD1 repression (n = 3 in triplicate, mean ± SEM).

Based on the above observations, we investigated the role of EEPD1 in the major DNA DSB repair pathways by using two previously described assays. EEPD1 depletion increased cNHEJ by 2.3-fold in the EJ5 cell reporter system (Fig 3A) [37,38], implying that EEPD1 inhibits cNHEJ. EEPD1 depletion reduced HR repair of I-SceI induced DSBs by 6.4-fold in the HT256 reporter system (Fig 3B) [39]. This reduction in HR raised the question of whether EEPD1 depletion increased gene conversion tract lengths. Cells with defects in HR components display longer gene conversion tracts among residual HR products [30,31,40–46]. Consistent with these prior studies, HR products from EEPD1-depleted cells had significantly longer conversion tracts compared to controls (Fig 3C and 3D). The longer gene conversion tracts are thought to reflect unstable heteroduplexes [40] and/or defective resection preventing efficient 5’ end-capture by the invaded template [30,46]. In the case of EEPD1 depletion, we hypothesize that defective end resection (shown below) results in less efficient 5’ end-capture. If true, then this implies that less efficient SS end-capture reactively stimulates synthesis along the invaded template, an idea supported by several published reports [30,31,42].

Cells with HR defects, such as those with BRCA1 or BRCA2 mutations, are hypersensitive to PARP1 inhibitors, due to an increase in unrepaired DSBs arising during replication [47–49]. We therefore repressed EEPD1 in BRCA1/2 proficient cells and assessed the effect of the PARP1 inhibitor olaparib on cell survival. EEPD1 repression markedly increased the cytotoxicity of olaparib (19-fold, Fig 3E), in the absence other genotoxins, consistent with EEPD1 playing a significant role in HR repair.

**Fig 4:**
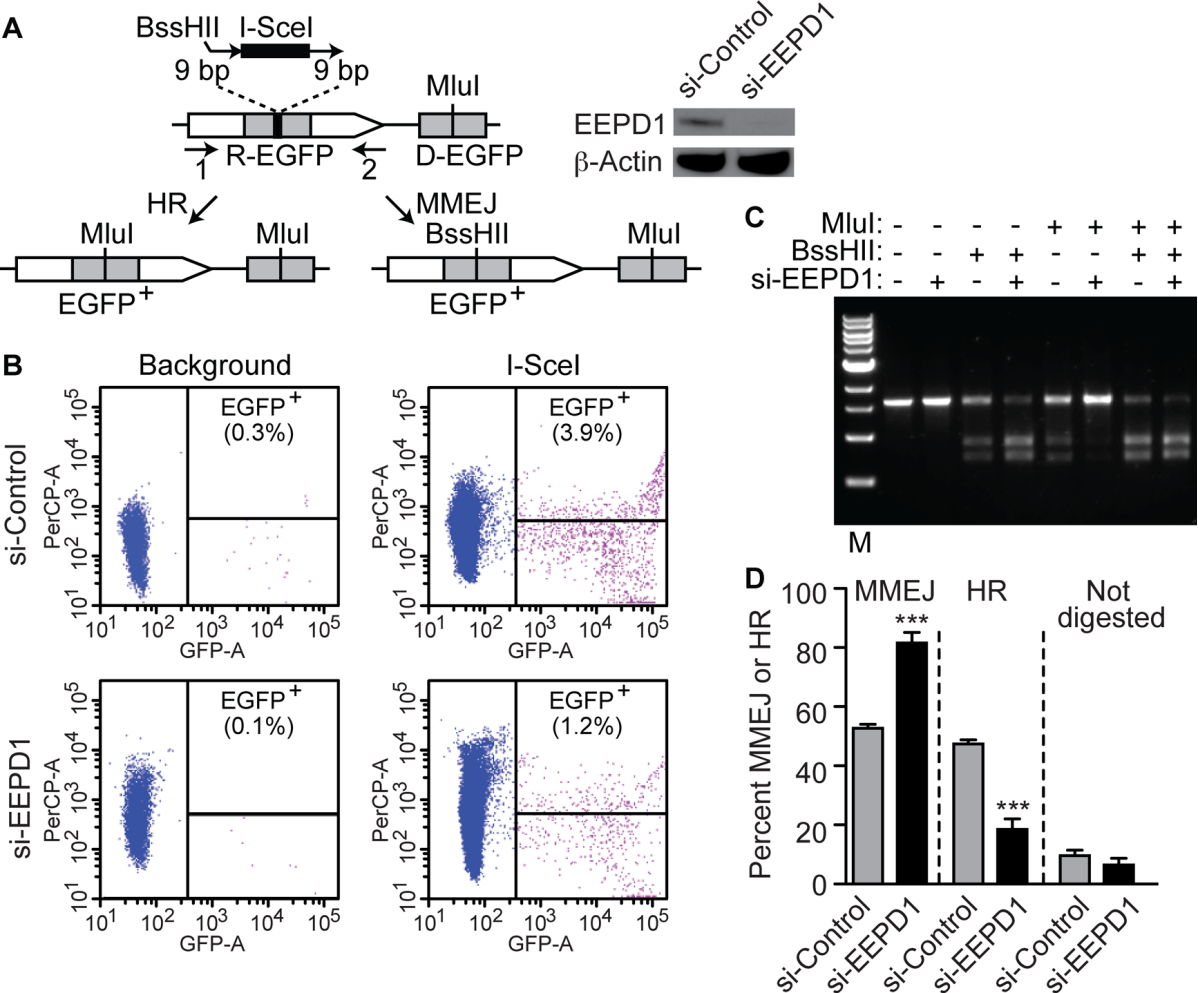
HR requires EEPD1 to a greater extent than MMEJ. (A) Schema of the MMEJ/HR-MluI reporter system and Western blot confirming EEPD1 knockdown. (B) Flow cytometry of cells with and without I-SceI transduction. (C) Representative results of PCR amplified EGFP^+^ products digested with BssHII (MMEJ) or MluI (HR). The percentage of the total product digested by each enzyme indicates the relative utilization of each repair pathway. (D) Graphical representation of the densitometric analysis of the cleaved PCR products over total products, showing relative fractions of HR and MMEJ in cells with or without EEPD1 depletion (n = 3).

There are two DSB repair pathways that use 5’ end resection to initiate the repair cascade, microhomology-mediated end joining (MMEJ), and HR. The frequency of utilization of these two pathways can be compared at a single induced DSB in the EGFP-based MMEJ/HR-Mlu1 reporter (Fig 4A) [19]. Upon DSB induction with I-SceI transduction, repair by either MMEJ or HR results in loss of the I-SceI site and generation of EGFP, allowing repaired cells to be sorted by flow cytometry (Fig 4B). The repaired EGFP loci were PCR amplified, and analyzed for repair by HR versus MMEJ. Cells repaired by MMEJ have a 9 nt duplication containing a BssHII site, while cells repaired by HR have an MluI site (Fig 4C). The fraction of BssHII cleaved products among the total PCR products represent the fraction repaired by MMEJ, while the fraction cleaved by MluI represents HR repair.

Depletion of EEPD1 resulted in an average decrease of 2.5-fold in EGFP-positive cells in the MMEJ/HR-MluI reporter system. The EGFP locus was PCR amplified from EGFP-positive cells, and digested with BssHII or MluI. This revealed that EEPD1 depletion resulted in a 9-fold reduction in HR and a 50% increase in MMEJ (Fig 4D). This implies that when MMEJ and HR are competing at a single DSB site, EEPD1 pushes that repair decision towards HR, and away from MMEJ. This may mean that EEPD1 is important for initiating long range end resection used in HR, consistent with its interaction with Exo1/BLM as noted below.

### EEPD1 Promotes End Resection

**Fig 5:**
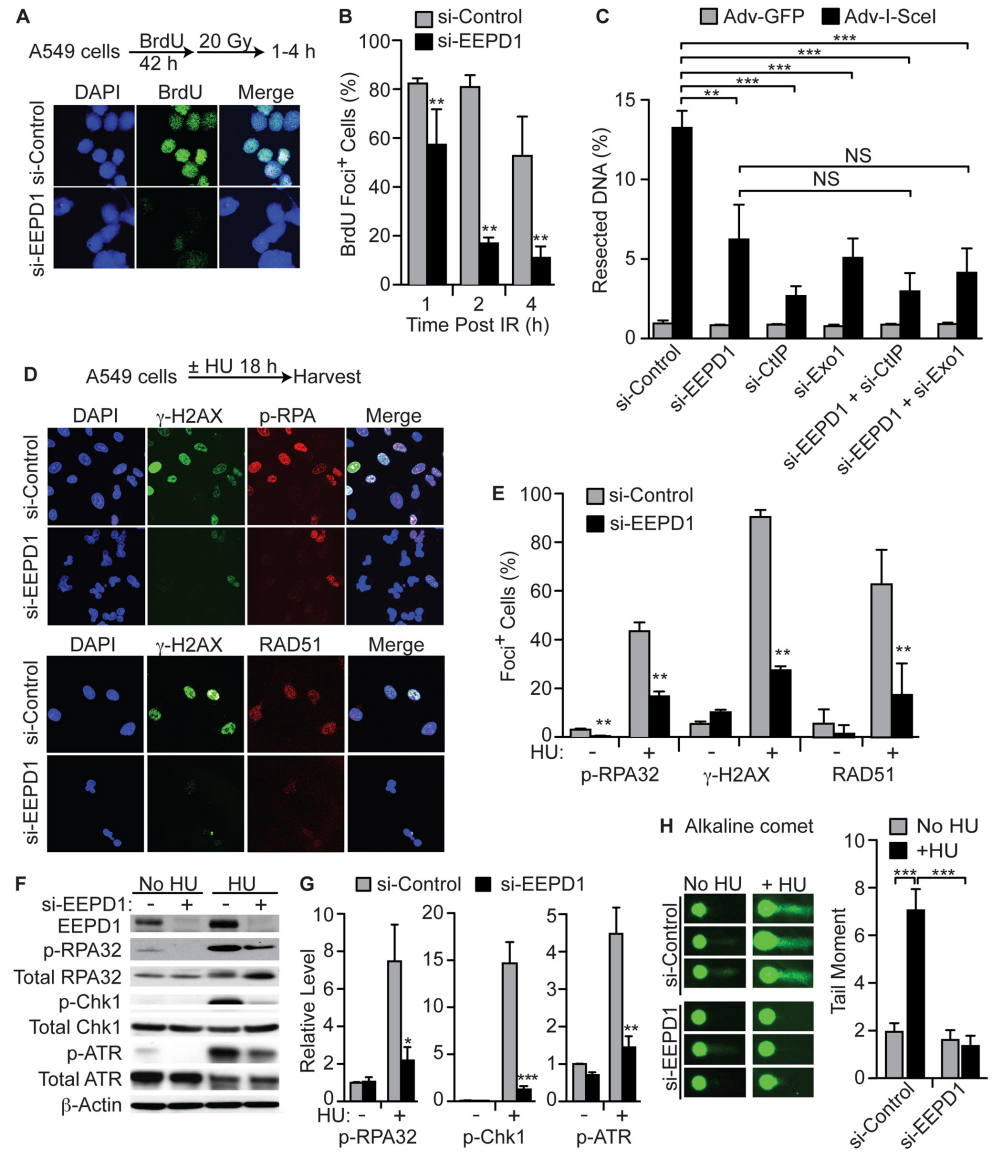
EEPD1 promotes end resection and downstream replication stress signaling. (A,B) End resection after IR measured by the fraction of cells with ss BrdU present in non-denatured DNA. Representative images (A) and quantitation (B) are shown as mean ±SD, n = 11–19 per condition, 139–180 cells/condition. (C) End resection adjacent to a single I-SceI DSB in HT1904 cells was measured in control cells and in cells depleted for EEPD1, CtIP and/or Exo1, alone or together (n = 3, means ± SD). For depletion of each protein, see S6 Fig. (D,E) Phospho-S4/S8 RPA32, gamma-H2Ax, and RAD51 nuclear foci formation in A549 cells after replication stress with and without EEPD1 depletion. DAPI nuclear counterstain is blue. (F,G) Phosphorylation of ATR (T1989), Chk1 (S345), and RPA32 (S4/S8) analyzed by Western blot in HU or mock-treated A549 cells with and without EEPD1 depletion. Representative blot (F) and quantification (n = 3–4 blots, means ±SD) (G) presented as relative protein levels normalized to β-actin loading controls. (H) Representative results (left) of alkaline single cell electrophoresis assays in untreated or HU-treated A549 cells with or with EEPD1 depletion, and quantitation (right) showing means ±SEM (n = 5).

Since the key step for determining DSB and replication fork repair pathway choice is 5’ end resection [16–18], we therefore assessed the role of EEPD1 in 5’ end resection after DSB formation using two approaches. First, we measured the generation of SS DNA at IR-induced DSBs by immunostaining newly incorporated BrdU in non-denatured SS DNA [50,51]. We found that depletion of EEPD1 reduced the number of cells with SS BrdU after IR by 5-fold (Fig 5A and 5B). In the second approach, we assessed resection around an induced I-SceI DSB [52,53]. Using this technique, we found that EEPD1 depletion reduced end resection by 3-fold, nearly the same extent as CtIP depletion (Fig 5C). Thus, EEPD1 is important for end resection after both a transduced restriction enzyme (I-SceI) and exogenous IR.

We next tested whether EEPD1 functions in the same end resection pathway as Exo1 or CtIP (Fig 5C). We depleted EEPD1 with or without co-depletion of Exo1 or CtIP. There is no significant difference in end resection when EEPD1 and Exo1 are co-depleted compared to individual depletion, suggesting that these enzymes function in the same resection pathway. Co-depletion of EEPD1 and CtIP also yielded similar results as individual depletions. These results suggest that EEPD1 functions in the same resection pathway(s) as Exo1 and CtIP.

### EEPD1 Is Required for Loading DNA Repair Components onto Damaged Replication Forks

When a replication fork collapses, SS DNA arises by end resection, or by uncoupling of the polymerase complex from the helicase [10,54,55]. Such ss DNA is coated by RPA, which recruits ATRIP, leading to ATR activation and phosphorylation of RPA, H2Ax, and Chk1 which mediate cell cycle arrest and replication fork repair [56–58]. To define the epistatic position of EEPD1 in HR, confocal immunofluorescence microscopic studies of fork repair components were performed in cells treated with HU for a prolonged period, which causes replication fork collapse. We found that EEPD1 depletion significantly decreased foci formation by RPA32 (2.7-fold), gamma-H2Ax (3.3-fold), and RAD51 (3.5-fold) (Fig 5D and 5E). The decrease in RPA32 foci was consistent with its decreased phosphorylation, detected by Western blotting (Fig 5F and 5G). Consistent with the decreased formation of RPA32, gamma-H2Ax, and RAD51 foci, and the requirement for SS DNA to trigger ATR and Chk1 signaling, EEPD1 repressed cells also showed decreased phosphorylation of ATR (3-fold) and Chk1 (11-fold) (Fig 5F and 5G). EEPD1 did not co-localize with gamma-H2Ax foci after damage (S3 Fig), not surprisingly, since EEPD1 appears to act upstream of gamma-H2Ax.

### NBS1, 53BP1 and BRCA1 Foci Are Intact in EEPD1-Repressed Cells

The Mre11-Rad50-NBS1 (MRN) complex is a first responder to DSB damage [59–61]. NBS1 recruits BRCA1 to stalled replication forks in an alternative pathway to the canonical gamma-H2Ax/MDC1/RNF8/BRCA1 recruitment pathway [62,63]. Confocal immunofluorescence studies were performed to investigate whether EEPD1 depletion impairs these early regulators of DSB repair. While HU-induced RPA32, gamma-H2Ax, and RAD51 foci were significantly decreased in EEPD1 depleted cells (Fig 5D and 5E), BRCA1, 53BP1, and NBS1 foci were unaffected (S3 Fig), indicating the initial 53BP1 recruitment step of cNHEJ was functional, and that NBS1 and BRCA1 have upstream roles in repair of stressed forks. These data imply that MRN/BRCA1 and EEPD1 act in distinct repair pathways.

For 5’ end resection to take place at a stalled replication fork, there must be a free DNA DSB end [3,4,8–12]. This can occur via fork reversal to form a chicken foot structure, but the majority of stalled forks do not reverse [20]. For those stalled forks that do not reverse the fork must be nicked to create this required free DNA DSB end [3,13,14,17]. If this is true, then DNA nicking should be increased after HU nucleotide depletion to stall replication forks. We assessed the occurrence of DNA nicking using alkaline single cell electrophoresis assays with and without EEPD1 depletion (Fig 5H). We found that after HU exposure, DNA nicking increases 3.5-fold. However, EEPD1-depletion completely abolishes this increase, implying that EEPD1 is directly or indirectly responsible for DNA nicking in response to HU-induced replication stress.

EEPD1 is expressed primarily in the nucleus, consistent with it functioning as a nuclease (S4 Fig). Since EEPD1 has homology to RuvA, which binds to heteroduplex chicken foot structures, and it has a nuclease domain, we examined whether EEPD1 has nucleolytic activity on chicken foot structures. We found that recombinant EEPD1 protein did not nick any of the four double-stranded regions of the regressed fork, but it does have specific 5’ overhang endonuclease activity (S5 Fig), cleaving at a single site at the joint of the overhang. Chicken foot structures with 5’ overhangs are difficult for Exo1 to process [64]; EEPD1 could promote further 5’ end resection by presenting Exo1 with a more amenable structure. These data demonstrate that EEPD1 is a 5’ endonuclease, consistent with the marked reduction in HU-induced nicks in EEPD1 depleted cells.

We next assessed the effect of EEPD1 depletion and co-depletion of three other resection components, CtIP, Exo1, and Dna2, on cell proliferation with or without HU-induced replication stress (S6 Fig). EEPD1 depletion alone suppressed cell proliferation, as did depletion of CtIP and Dna2. Co-depletion of EEPD1 with CtIP or Dna2 did not further affect proliferation, with or without replications stress. By contrast, Exo1 depletion only modestly suppressed proliferation, and only with replication stress. Again, there was no further effect on proliferation with co-depletion of EEPD1 and Exo1 than with EEPD1 depletion alone.

We also compared the effects of EEPD1, CtIP, Exo1, and Dna2 depletion singly and in pairs on the formation and resolution of after HU-induced replication stress (S7 Fig). As above (Fig 5D and 5E), EEPD1 depletion strongly suppressed the formation of gamma-H2Ax foci 4 and 24 h after HU, with similar or greater effects than CtIP depletion. Co-depletion of EEPD1 and CtiP did not further suppress gamma-H2Ax foci 4 h after HU. Exo1 depletion had similar effects as CtIP depletion, with or without co-depletion of EEPD1. Dna2 depletion enhanced gamma-H2Ax focus formation, even in the absence of replication stress, indicating that Dna2 plays a key role in preventing endogenous DNA damage. Co-depletion of Dna2 and EEPD1 did not significantly suppress gamma-H2Ax foci in untreated cells or after HU exposure. The enhanced gamma-H2Ax foci with co-depletion of EEPD1 and CtIP may also reflect enhanced or more persistent DNA damage caused by the genomic lesions, independently of induced replication stress.

### EEPD1 Is Recruited to Stalled Replication Forks

**Fig 6:**
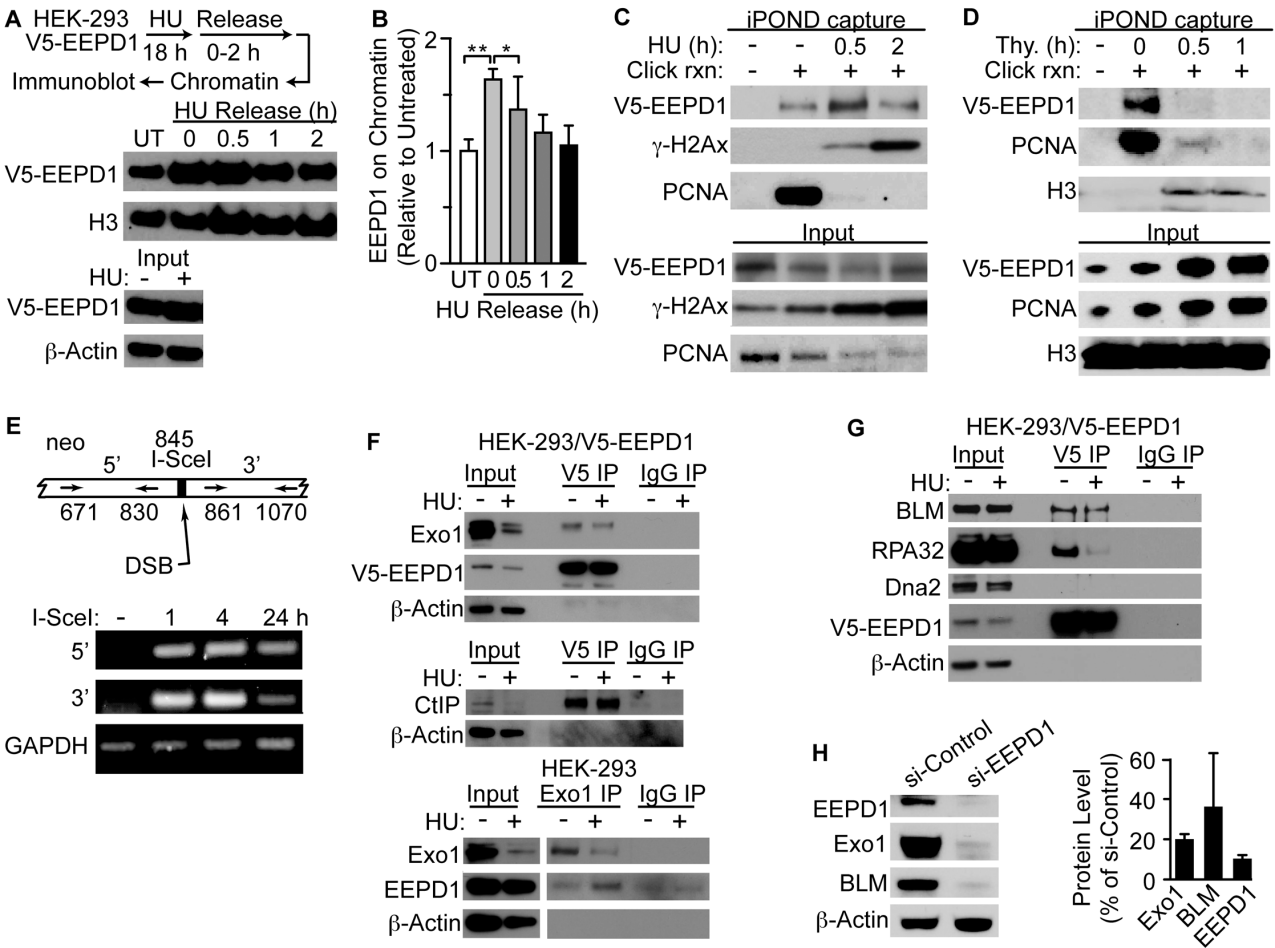
EEPD1 is recruited to replication forks in response to HU replication stress. (A,B) HEK-293 cells over-expressing wild-type V5-tagged EEPD1 treated with 10 mM HU for 18 h, chromatin was isolated 0–2 h after HU release and probed for EEPD1, and histone H3 as loading control (n = 4). Immunoblots (A) and densitometric measures of EEPD1 (B) are shown as average relative protein levels (means ±SD, n = 4) normalized to H3 as a chromatin loading control. (C) iPOND analysis of HEK-293 cells over-expressing V5-tagged EEPD1. Cells were incubated for 10 min in medium with 10 uM EdU to label nascent DNA, and then treated with 3 mM HU for indicated times to stall replication forks. (D) Control iPOND assay using a thymidine chase confirms the specificity of EEPD1 recruitment to nascent DNA. (E) Chromatin immunoprecipation of EEPD1 recruited to single DSB within neo locus in HT1904 cells. Schema showing PCR primer pairs relative to DSB site (above) and PCR results (below). (F,G) Co-immunoprecipitation of EEPD1 with Exo1, CtiP, BLM, and RPA32, but not Dna2. (H) Degradation of Exo1 and BLM when EEPD1 is depleted. Representative blot above, quantitation (mean ±SEM) of three replicate blots, below.

Western analysis revealed that after replication stress with HU, EEPD1 is enriched in the nuclear chromatin fraction (Fig 6A and 6B), suggesting that EEPD1 is recruited to chromatin containing damaged replication forks. We next assessed EEPD1 recruitment to stalled replication forks using Isolation of Proteins on Nascent DNA (iPOND) [65,66]. iPOND showed that EEPD1 is recruited to replication forks within 30 min of HU treatment, coinciding with the appearance of gamma-H2Ax (Fig 6C) which marks DSBs at stalled/collapsed replication forks [65]. PCNA was absent from the HU-stalled forks, consistent with replisome unloading from collapsed fork Okazaki fragments [65]. A control iPOND assay using a thymidine chase confirmed the specificity of EEPD1 recruitment to stalled forks (Fig 6D). By using chromatin immunoprecipition [52] we also demonstrated that EEPD1 is recruited to an I-SceI induced DSB (Fig 6E). Interestingly, EEPD1 constitutively co-immunoprecipitates with Exo1, RPA32, and BLM in the presence of DNase, whether or not replication stress is present, indicating that these proteins reside in the same complex (Fig 6F and 6G). However, EEPD1 does not co-immunoprecipitate with Dna2, indicating that it is likely not in the RPA/Dna2/MRN end resection complex [28]. Significantly, depleting EEPD1 reduced Exo1 and BLM protein levels, suggesting that EEPD1 promotes stability of the complex in which EEPD1, Exo1, and BLM reside (Figs 6H and S6B), indicating that EEPD1 resides in an obligate complex, perhaps to prevent aberrant nuclease activation and improper DNA cleavage.

### EEPD1 Maintains Genome Stability

**Fig 7:**
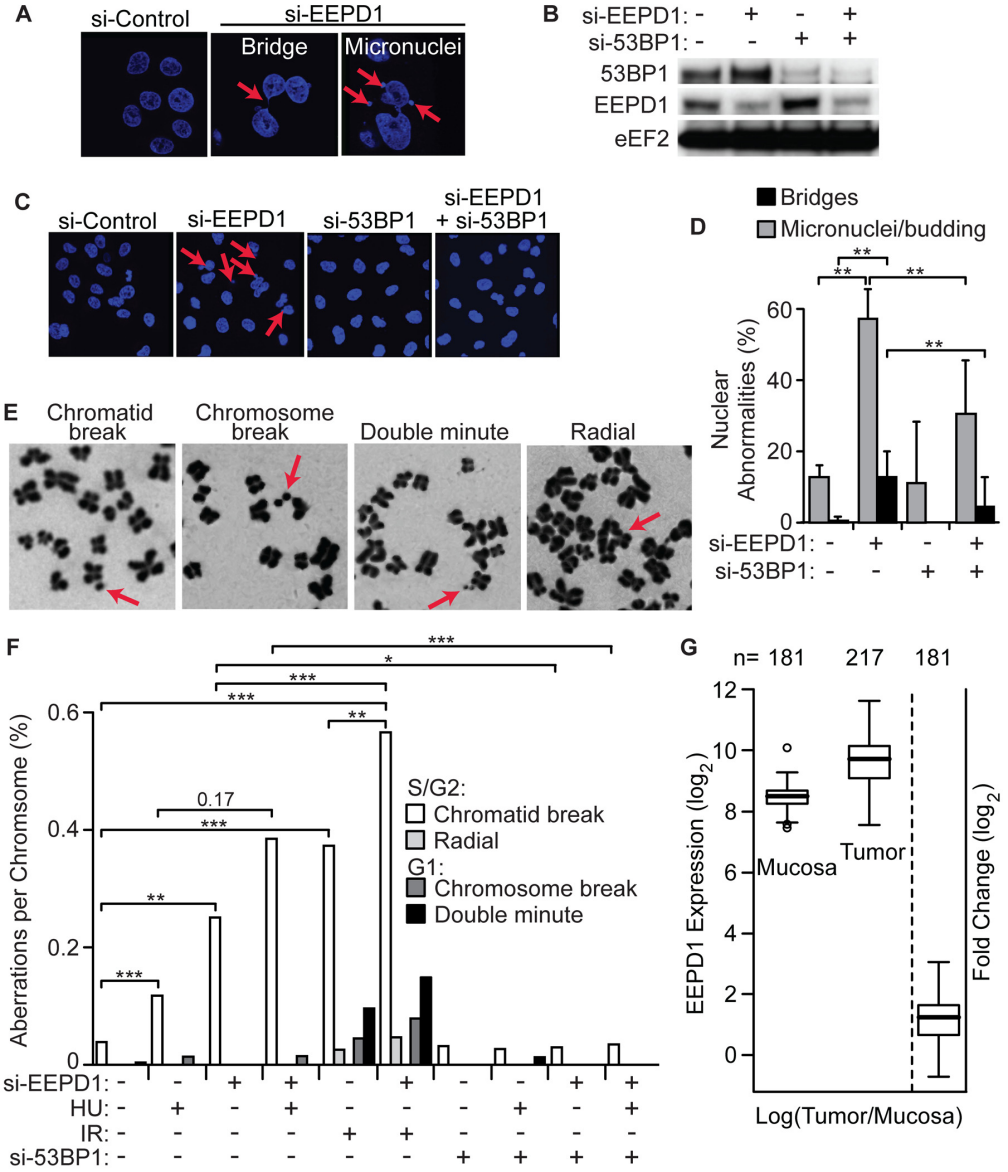
EEPD1 maintains genome stability and is overexpressed in colorectal cancers. (A) A549 cells were transfected with control siRNA or si-EEPD1 were stained with DAPI and analyzed for nuclear aberrations. Arrows indicate micronuclei and nuclear bridges. (B) Confirmation of 53BP1 and/or EEPD1 depletion by Western blot analysis of A549 cells transfected with cognate siRNAs; loading control is eEF2. (C,D) Representative images of unstressed A549 cells from panel B, with arrows indicating micronuclei (C), and quantitation of nuclear bridges and micronuclei plotted as mean percentages of nuclear aberrations (n = 10, 142–190 nuclei/determination) ± SD. (E) Representative photomicrographs of chromosome aberrations. (F) Quantification of chromosome aberrations in A549 cells treated with HU, IR or untreated, and with depletion of EEPD1 and/or 53BP1 (n = 3 metaphase spreads per condition, 102–374 metaphases scored per spread). (G) EEPD1 expression was determined in colorectal carcinomas and adjacent normal mucosa samples. Box and whisker plots are shown with median (heavy line) and upper/lower quartiles indicated (bars). EEPD1 expression is 2.3-fold higher in tumor samples (P = 9×10^−30^).

Proper replication stress responses are required to prevent gross chromosomal instability, which can be assessed by the formation of micronuclei and nuclear bridges that result from mis-segregation of fused chromosomes [27,67]. We found that EEPD1-depleted cells display severe nuclear anomalies, with 6- and 7-fold increases in nuclear bridges and micronuclei, respectively (Fig 7A–7D). Chromosome fusion events occur when collapsed forks are aberrantly repaired, as in BRCA1-deficient cells with unopposed 53BP1 [22,23]. 53BP1 depletion alone did not alter nuclear anomalies, but 53BP1 depletion largely suppressed both bridges and micronuclei associated with EEPD1 depletion.

Metaphase analysis further demonstrated EEPD1 repression causes genome instability, revealed as significant increases in chromatid breaks and radial chromosomes, both of which arise in S/G2 cells (Fig 7E and 7F). Interestingly, IR induced these S/G2-associated events, and at lower frequencies, G1-associated chromosome breaks and double minutes. EEPD1 depletion alone did not increase G1-associated events, nor did it affect the frequency of IR-induced these events (Fig 7F). Thus, EEPD1 specifically suppresses S/G2 events. Although more chromatid breaks were observed in EEPD1-depleted cells treated with HU than with HU alone, the difference was not significant (P = 0.17); EEPD1 depletion did significantly increase IR-induced chromatid breaks compared to IR alone. Interestingly, 53BP1 repression fully suppressed spontaneous and HU-induced chromatid breaks seen in EEPD1 depleted cells as well as HU-induced chromatid breaks in cells with normal EEPD1 expression (Fig 7F). These results indicate that EEPD1 plays a critical role in maintaining genome stability, under stressed and non-stressed conditions, and suggest that EEPD1 promotes genome stability by mediating accurate HR repair of stressed replication forks.

### EEPD1 Is Frequently Overexpressed in Colorectal Cancer

Cancer cells experience continuous replication stress due to metabolic alterations and checkpoint defects that permit DNA replication despite significant DNA damage. To manage this stress, it is reasonable to suppose that EEPD1 would be up-regulated in cancers. We tested this by analyzing mRNA expression of EEPD1 in newly resected colorectal cancer versus adjacent normal tissue. In the present study we analyzed EEPD1 expression in 181 new colorectal cancers, and found that EEPD1 was expressed an average of 2.3-fold higher than adjacent normal tissue in 171 of 181 cases (Fig 7G).

## Discussion

This study demonstrates that the uncharacterized EEPD1 nuclease plays a key role in repairing stressed replication forks via HR. Interestingly, while EEPD1 confers resistance to replication stress, it only appears to accelerate fork restart by 10 min. Thus, nearly all forks still restart within 30 min of release from stress in EEPD1-depleted cells compared to 20 min in wild-type cells (Fig 2B and 2C). These results imply that when fork repair is repressed, even a relatively brief delay in fork restart can be lethal, perhaps because toxic recombination intermediates form if stalled forks fail to restart in timely manner [12,32,35]. EEPD1 is also important under non-stress conditions, as EEPD1 depletion significantly slows cell growth rate. Thus, EEPD1 probably promotes restart of replication forks that encounter DNA lesions arising spontaneously during normal cellular metabolism.

There are numerous reports demonstrating that most cells do not tolerate long delays in the restart of stalled replication forks, if repair is impaired. BRCA1 deletion results in cell death after even a brief period of replication stress [68]. There is evidence that forks blocked by interstrand crosslinks are restarted via lesion by-pass long before the lesion is repaired [69]. Thus, restarting replication forks appears to be a higher priority for the cell than repair, at least in some situations. When an ATR inhibitor is combined with a fork stalling agent, cells in S-phase lose all ability to recover within 45 min [70]. Removing the replication stalling agent and ATR inhibition after that point does not restore cell viability. Reintroduction of the DNA damage checkpoint in yeast mutants after a brief period of replication stalling does not rescue cell viability [71]. Our data here show that the largest effect of EEPD1 on stalled fork repair and restart is 10 min after HU release, yet EEPD1 is required for survival to many replication stress agents. Thus, when replication fork repair is impaired, even a brief period of fork stalling can be lethal.

Interestingly, cells proliferate more slowly when EEPD1 is depleted. In addition, even in the absence of replication stress with HU, cells with depleted EEPD1 have increased gamma-H2Ax and decreased RPA foci. Thus, cells lacking EEPD1 appear to experience spontaneous replication stress. This may be due to EEPD1 having a role in normal replication fork progression, perhaps in nucleolytic processing of replication fork lagging strand intermediates [1–3,13,14,16,17]. A not mutually exclusive alternative is that EEPD1 promotes restart of forks stalled by spontaneous lesions, which may arise frequently in rapidly growing cultured cells. This would require constant repair of replication forks stalled by the continuously generated DNA lesions within their paths, which would be reliant on EEPD1.

EEPD1 represses cNHEJ and enhances HR rates significantly. This indicates that EEPD1 plays an important role in DNA DSB pathway choice. By promoting 5’ resection EEPD1 would direct repair away from cNHEJ and towards resection-dependent repair pathways, namely HR and MMEJ [3,8–10,12,16,17,19]. PARP1 competes with the Ku complex for free DNA DSB ends to promote MMEJ over cNHEJ [72]. PARP1 inhibition with olaparib was synthetic lethal with EEPD1 depletion. This implies that EEPD1 depletion is not epistatic with PARP1 in replication fork repair or in MMEJ repair [49,73], given that there is additional cytotoxicity when both are repressed.

Both HR and MMEJ require 5’ end resection to begin their repair cascades [17,19]. Both pathways can repair and restart replication forks after stalling [19]. Recent reports indicate that DNA polymerase (pol) theta may suppress HR and promote MMEJ repair of DNA breaks [74]. HR-deficient tumors rely on pol theta for DNA DSB repair [75]. Pol theta enhances MMEJ by tethering free DSB ends after short range end resection for the microhomology search [76]. EEPD1 would seem to have the opposite effect, promoting HR at the expense of MMEJ (Fig 4). This would be beneficial because unopposed pol theta−mediated MMEJ repair of replication forks would increase non-conservative repair, and more importantly, chromosomal fusions [74,75]. It is possible that EEPD1 promotes HR over MMEJ by enhancing long range end resection [17,19,30,31,76], perhaps via its interaction with Exo1. Exo1 seems to be important for long range end resection during HR [17,19,77].

End resection promoting HR at DSBs is thought to initiate when BRCA1/CtIP displaces Rif1/53BP1 at DSBs [23–26]. CtIP has an important non-nuclease role in initiating 5’ end resection at undamaged DSB ends. CtIP may also have nuclease activity important for resection of damaged DSB ends, but this is controversial [24,30,60,78]. Thus, in addition to its role in cleavage of stressed replication forks, EEPD1 may also be important for initiating end resection at undamaged DSB ends in HR, where CtIP may not have a role. While the competing activities of Rif1/53BP1 and BRCA1/CtIP determines cNHEJ vs HR repair pathway choice, less is known about the role of these components in the repair decisions at stalled replication forks. The data here implies that EEPD1 directs the cell away from cNHEJ towards HR (Fig 3A and 3B), probably by enhancing end resection. EEPD1 functions in a distinct end resection pathway from BRCA1/CtIP, or downstream of BRCA1/CtIP, since HU-induced BRCA1 foci are intact in EEPD1-depleted cells (S3 Fig).

Replication forks require a free DNA end with which to initiate 5’ end resection for repair by either HR or MMEJ [8,10,13,16,20]. For approximately one quarter of replication forks stalled with HU, this occurs via fork reversal, with Dna2 then mediating 5’ end resection [20,79]. However, the majority of replication forks require a nick in one of the parent strands at the replication fork itself to create a free DNA end. HU-induced replication stress results in rapid DNA nicking, which is mediated by EEPD1 (Fig 5H). Given that EEPD1 is rapidly recruited to stalled replication forks, promotes 5’ end resection, HR, and replication fork restart, it is possible that the lack of DNA nicking seen after EEPD1 depletion is due to the failure of replication fork cleavage. This failure to cleave the stressed fork may prevent 5’ end resection for HR-mediated fork repair and decrease fork restart. This implies that some stressed replication fork nicks, rather than contributing to cell death, may instead promote cell survival by accelerating fork restart, perhaps by preventing accumulation of toxic HR intermediates [4,6]. Since many stalled replication forks do not reverse to form a one sided DNA free end for end resection [20], such cleavage is often necessary to initiate end resection and HR [8,10,13,16].

Placing the various end resection nucleases epistatically within the context of 5’ end resection is challenging [13,14,17]. From a biochemical standpoint, there appear to be two end resection complexes, BLM-DNA2-RPA-MRN and EXO1-BLM-RPA [28], with EEPD1 as a component of the latter. However, there is functional overlap between these complexes, and both are likely essential for HR and cell survival in response to replication stress [16,18]. From the data presented here, there is little additional deficiency in end resection after replication stress when EEPD1 is doubly depleted with Exo1 or CtIP. Interestingly, Dna2 depletion increases gamma-H2Ax formation de novo and after replication stress, while Exo1 and EEPD1 depletion reduce gamma-H2Ax formation after replication stress. Thus, Dna2 appears to operate downstream of gamma-H2Ax signaling, while EEPD1/Exo1 are upstream, but perhaps both complexes are needed to repair distinct forms of stressed replication forks [20].

End resection creates SS DNA that can signal replication stress and cell cycle arrest. EEPD1 depletion abrogates gamma-H2Ax foci (Fig 5D and 5E), indicating resection promoted by EEPD1 precedes phosphorylation of H2Ax during replication fork repair. Similarly, ATR is activated by RPA/ATRIP loading onto SS DNA, ultimately activating Chk1. There are two possible explanations for the finding that EEPD1 is required for ATR/gamma-H2Ax/Chk1 phosphorylation after HU. First, the resection defect in EEPD1-depleted cells may account for a fraction of the ATR and Chk1 activation defects after replication stress. In this scenario the SS DNA created by end resection plays a key role in RPA/ATRIP activation of ATR. Second, the SS DNA that signals ATR activation may arise not from end resection but from the disassociation of the helicase from the polymerase complex [54], and in this case EEPD1 might play a role in RPA/ATRIP signaling to ATR. In either case, at least for replication stress induced by HU, EEPD1 is an important factor in the activation of ATR/Chk1.

Interestingly, EEPD1 is in a constitutive and obligate complex with Exo1 and BLM (Fig 6F and 6G). The long resection exonuclease Exo1 requires a free 5’ DNA end to initiate resection at a damaged fork. Since EEPD1 and Exo1/BLM constitutively co-immunoprecipitate, this implies that EEPD1 is a partner in the Exo1/BLM/RPA end resection complex [28]. It also implies that when EEPD1 is recruited to the damaged fork, it is accompanied by Exo1/BLM, which are needed for completion of end resection. The obligate nature of this complex is not surprising, since nuclease function must be exquisitely balanced to prevent wide-spread and unregulated genome incision. It is imperative the cell tightly control all nucleases to prevent inappropriate or untimely DNA cleavage to suppress translocations, and to maintain genome stability.

Our results also indicate that EEPD1 helps maintain genome stability. As proposed for cells with defects in other HR proteins like BRCA1 and BRCA2 [21–23], the genome instability seen in EEPD1 depleted cells is likely a direct consequence of unopposed cNHEJ causing aberrant ligation of DNA ends at distinct collapsed replication forks. This hypothesis is supported by the fact that genome instability observed in EEPD1 depleted cells is suppressed by depletion of the cNHEJ promoting factor 53BP1 (Fig 7A–7F). There is another potential reason why EEPD1 may prevent chromosomal instability- The MMEJ pathway mediates chromosomal translocation events when replication stress overcomes the ability of the cell to repair such stress [74,75,77] EEPD1 promotes HR over MMEJ (Fig 4) and this could suppress chromosomal instability during replication stress by directing fork repair toward HR which is less prone to chromosomal fusions. Thus, EEPD1 may prevent chromosomal translocations by promoting HR and suppressing MMEJ during repair of chromosomal DSBs. Our results indicate that EEPD1 is an important guardian of genome stability that functions by regulating replication fork repair pathway choice.

Although the mechanism by which EEPD1 depletion sensitizes cells to replication stress is not well defined by the present study, many prior studies that demonstrate HR defects increase sensitivity to replication stress correlate increased sensitivity with increased gamma-H2Ax [reviewed in refs.1,2,3,8]. While it is widely accepted that stressed replication forks can collapse into aberrant structures in cells lacking HR machinery to repair them [1,13,14,19], whether these aberrant structures actually cause cell death is not known. We demonstrate here that EEPD1 depletion increases cell death in the face of replication stressors, and that it reduces HR, slows replication fork restart, reduces DNA nicking, and creates cytogenetic and nuclear abnormalities. The marked increase in micronuclei and mitotic bridges in EEPD1 depleted cells is exacerbated by replication stress, and this suggests the following mechanism for cell death from EEPD1 deficiency during replication stress: collapsed replication forks end-join aberrantly, creating chromosomal fusions that are manifest as mitotic bridges and micronuclei. These gross chromosome abnormalities would intuitively be difficult for cells to recover from, and therefore may serve as a better correlation between HR and cell death than gamma-H2Ax.

One would predict that malignancies would require intact EEPD1 to proliferate, and that loss of function mutations would be rare in human cancers. This is indeed the case; there are only 31 coding changes in EEPD1 out of 8273 individual cancer genome sequences in the COSMIC database (http://cancer.sanger.ac.uk/cosmic), and the vast majority of these are conservative, and are not predicted to alter function. This is not surprising, as a malignancy with EEPD1 functional loss would have difficulty proliferating given that tumors often survive despite significant replication stress caused by oncogene activation, hypoxia, and/or nutrient deprivation [6]. Thus, even though loss of EEPD1 results in genomic instability, EEPD1 should not be viewed as a tumor suppressor in the same sense as BRCA1 and BRCA2, two HR components that show loss of function mutations in cancer. It is likely that loss of EEPD1 function would be too detrimental to replication fork restart and fork progression to be selected for during oncogenesis, because of the fundamental importance of DNA replication to malignant cells.

On the other hand, given its role in replication fork rescue, EEPD1 could be an excellent target for treatment of human malignancies. EEPD1 is over-expressed in nearly all colorectal cancers [80] (Fig 7G) and large cell lymphomas [81], cancers whose treatment is based on agents that create replication stress. Targeting EEPD1 could block proliferation of cancers that depend on EEPD1, or sensitize tumors to chemotherapeutics that cause replication stress. Such agents are the foundation for treating both of these types of malignancies [81–83].

## Materials and Methods

### Cell Culture, Transfection and Survival Assays

A549, HEK-293, HEK-293T, HT256 reporter cells, and the various U2OS reporter cells (EJ5, MMEJ/HR), were cultured in D-MEM supplemented with 10% fetal bovine serum and 1% penicillin and streptomycin. HT256 cells were cultured in Alpha-MEM supplemented with 10% fetal bovine serum and 1% penicillin and streptomycin. EEPD1 was depleted using two mechanisms, shRNA and siRNA, to control for variation in the method of mRNA destruction. EEPD1 was depleted either by 1) EEPD1 lentivirus shRNAs produced from 293T cells (pLKO.1, Thermo Scientific, Pittsburgh, PA); or 2) SMARTpool ON-TARGETplus EEPD1 siRNA from Dharmacon RNAi Technologies (GGACUGACCUUCACCGCCA; CUGAGAAGCCCUCGAGUCA, GGAAGUUGACCUCGGGGUA; UGCGAGAGGUGGUGUGCAU) (Pittsburgh, PA). EEPD1 3’UTR On-Target plus siRNA also from Dharmacon (GGAAGUUGACCUCGGGUA). 53BP1siRNA(h) is a pool of 3 different siRNA duplexes from Santa Cruz Biotechnology (sc-37455). All other siRNAs were from Dhamarcon SMARTpools. All nucleic sequences are listed 5’ to 3’ in this Supplement.

Polyethylenimine (PEI) was used to perform plasmid transfections according to the manufacturer’s instructions (Thermo Scientific). Briefly, PEI was incubated with plasmid DNA at 3:1 ratio in Opti-MEM at RT for 20 min before addition to cells. After 6 h incubation, cells were washed and placed in fresh media. RNAiMAX (Invitrogen, Grand Island, NY) was used to transfect siRNA pools. Briefly, RNAiMAX was incubated with 50 nM of siRNA in Opti-MEM at RT for 20 min before addition to cells. After 24 h cells were washed and placed in fresh media. EEPD1 repression was confirmed by western blotting for every experiment. At least two clones were used for each Lentiviral shRNA experiment, to control for clonal variation in repression. There was no difference in phenotypes obtained between the shRNA and the siRNA repression of EEPD1.

Experiments were repeated using both techniques for EEPD1 depletion to control for off-target effects of the mechanism of repression. All experiments were performed at least three times, in at least two cell lines, to control for experimental and cell line variation. A549 lung cancer cells were used for most replicative experiments since this cell line has high EEPD1 expression. All studies in A549 were also repeated at least once in HEK-293 cells to control for cell lineage variability. Clonal survival after treatment with DNA damaging agents was determined by seeding 2,000 cells per 10 cm dish in either control media or media with varying concentrations of genotoxic chemicals, or exposure to varying doses of IR or UV light. Cells were exposed to etoposide or olaparib for 18 h, then washed and incubated in fresh media for 12 days. Colonies were stained with 0.1% crystal violet in methanol and counted. A colony greater than 50 cells was counted as a surviving clone. For HU, cells were treated continuously for 12 days before colonies were stained and scored. Plating efficiency was calculated as the number of colonies divided by the number of cells plated without genotoxin treatment. In all of these assays, survival was normalized to untreated cells transfected with control or EEPD1 si or shRNAs. Survival fractions were calculated as the number of colonies formed after exposure to a given genotoxin divided by the number of cells plated, then multiplied by the plating efficiency. Unpaired Student t tests were used for all statistical analysis, unless otherwise indicated. Each experiment was performed 6–9 times in triplicate.

### Western Blot Analysis and Antibodies

EEPD1 expression was monitored by standard western blotting protocol [36] using a custom-produced rabbit polyclonal antibody to EEPD1 peptide (CAEFYTEKDWSKKDAPRNHS, Lampire Biological Laboratories, Pipersville, PA). Phosphorylated RPA32 (S4/S8) and total RPA32 antibodies were from Bethyl Laboratories (Montgomery, TX). Phosphorylated ATR (T1989) antibody was from Genetex (Irvine, CA, cat. Gtx128145). Total ATR, phosphorylated Chk1 (S345), and total Chk1 antibodies were from Cell Signaling Technology (Danvers, MA). 53BP1 and BLM antibodies were from Abcam (Cambridge, MA), Exo1 antibody was from Proteintech (Chicago, IL), and beta-actin antibody was from Sigma-Aldrich (St. Louis, MO). When protein levels were quantitated, each western analysis was performed at least 3 times, with densitometric measures of band intensities normalized to loading controls. Student t tests were used for statistical analysis of the protein intensity differences.

### Immunoprecipitation and Chromatin Immunoprecipitation

Immunoprecipitation was performed with the Pierce Crosslink Magnetic IP/Co-IP kit according to manufacturer’s instructions (Thermo Scientific Cat.88805) as we described [36]. Briefly, HEK-293 cells overexpressing V5-tagged EEPD1 were treated, harvested and washed by PBS before lysis using IP lysis/wash buffer, then 5 ug of V5 mouse antibody (Invitrogen) were coupled to protein A/G magnetic beads and cross-linked with 20 uM disuccinimidyl suberate. The antibody cross-linked beads were incubated with cell lysate (0.8–1.2 mg) in a 500 ul of diluted lysate solution for 1 h at RT on a rotator. Beads were collected, washed and incubated with 100 ul of elution buffer for 5 min at RT. Antigen recovery was achieved by collecting the supernatant on a magnetic stand. Protease and phosphotase inhibitors were present in all buffers. ChIP was performed in HT256 cells using the procedure and GAPDH primers as we described [52]. ChIP primers for neo in HT256 152 nt from the I-SceI DSB site: Neo671 Forward: GACGGGCGTTCCTTGCGCAGCTG; Neo830 Reverse: CCAGATCATCCTGATCGACAAGAC. Primers 650 nt distant from the I-SceI site: Neo1 Forward: AAGCTTCACGCTGCCGCAAGCAC; Neo152 Reverse: GAACCTACCTGCTTTCTCTTTGC. GAPDH Forward: TCGGTTCTTGCCTCTTGTC; GAPDH Reverse CTTCCATTCTGTCTTCCACTC. Each immunoprecipitation was performed at least 3 times. Real time PCR to quantify immunoprecipitated sequences was performed using the SYBR green reagent (Applied Biosystems, Thermo Scientific) with the ABI 7000 sequence detection system, normalized to GAPDH amplification.

### Replication Fork Restart

Two methods were used for measuring stalled replication fork restart. In the first method, replication fork restart after arrest was measured by immunofluorescent detection of BrdU foci after DNA denaturation (BrdU in DS DNA), as we described previously [36]. Log phase A549 cells expressing normal or repressed levels of EEPD1, with or without expression of siRNA-resistant FLAG-tagged EEPD1 were incubated with 10 mM HU for 18 h and then released into media with 10 uM BrdU for 30 min. After washing, cells were fixed at different time points. Replication recovery was shown as percentage of cells with ≥ 3 BrdU foci 2h after release from HU. Cells without HU treatment served as controls for background staining from normal cell proliferation, which was used as threshold for measurement. Values are averages (± SEM) for 11–23 distinct determinations (>100 cells scored per condition).

The second method was DNA fiber analysis, as we previously described [32,35]. Both A549 and HEK-293 cells were tested to control for cell line differences. 600,000 cells were incubated overnight at 37°C in six-well plates. 20 mm IdU was added to growth medium and incubated for 20 min at 37°C. The IdU media was removed and cells washed in fresh medium, cells were treated with 5 mm HU for 60 min or mock-treated. The HU-containing medium was replaced with fresh medium containing 100 mm CldU. Cells were then incubated for varying times at 37°C. The CidU medium was removed, cells harvested, resuspended in PBS, and 1,000 cells were transferred to a positively charged microscope slide (Superfrost/Plus, Daigger), and processed for DNA fiber analysis as we described previously [32]. Slides were mounted in PermaFluor aqueous, self-sealing mounting medium (Thermo Scientific), and DNA fibers were visualized using a confocal microscope (Olympus, FV1000D, 63× oil immersion objective). Images were analyzed using the Olympus Fluoview software.

### DNA Damage Foci and Nuclear Structure Assays

Confocal immunofluorescence foci assays were performed as we described [35] with minor modifications. In brief, cells were cultured on coverslips followed by siRNA transfection and HU treatment. Cells were pre-extracted with 0.5% Triton X-100 and fixed with 4% paraformaldehyde for 20 min. Coverslips were then blocked with 1% BSA for 1 h before incubating with primary antibodies overnight. After washing twice, coverslips were incubated with secondary antibodies conjugated with Alexa Fluor dye (Invitrogen), mounted in anti-fade solution containing DAPI and stored at 4°C. All samples were analyzed within 24 h with a laser confocal scanning microscope (TCS-SP5, Leica Microsystems, Exton, PA). Cells with >5 foci were counted as positive. Photomicrographs of distinct cell populations were taken at equal magnifications and equal fluorescence intensities. For NBS1 and BRCA1 foci, the cells were fixed in 100% methanol and incubated with 1% BSA in 0.1% PBS-Tween for 1 h before incubating with primary antibodies overnight. RAD51 antibody was obtained from Santa Cruz Biotechnology (Dallas, TX). BRCA1 and RPA32 antibodies were obtained from Bethyl Laboratories (Montgomery, TX); gamma-H2AX (S139) antibody from Millipore (Billerica, MA), phosphorylated NBS1 (S343) and BrdU antibodies from Cell Signaling (Danvers, MA), and 53BP1 antibody from Abcam (Cambridge, MA). To assess nuclear structural abnormalities (micronuclei and post-mitotic bridging), control or HU-treated cells, with or without EEPD1 depletion, were grown on coverslips and fixed as above, and stained with 300 nM DAPI (Beckman) in PBS for 5 min. After washing thrice with PBS, coverslips were mounted in anti-fade solution and analyzed within 24 h. Of note, EEPD1 was located in the nucleus, but did not form discrete foci before or after damage. Each immunofluorescence assay was performed at least 3 times in triplicate.

### Isolation of Proteins on Nascent DNA (iPOND)

iPOND was performed as described by Sirbu and colleagues[65,66], with minor modifications to improve protein capture. In brief, HEK-293T cells over-expressing V5-tagged EEPD1 were seeded in three 150 mm plates/condition 24 h before the experiment. After 24 h incubation, 10 uM EdU (Invitrogen) was added to the medium for 10 min. EdU treatment was followed with 3 mM HU (Sigma, St. Louis, MO) at indicated times. The cells were then fixed with 1% formaldehyde (Sigma) for 10 min at RT, quenched by 0.125 mM Glycine (Sigma), and collected by scraping. The cells were permeabilized with 0.25% Triton X-100 for 30 min, and then subjected to click–iT reaction using Biotin azide (Invitrogen) for 90 min at room temperature. Lysis conditions were modified to reduce background: lysis was performed in 0.25% SDS lysis buffer for 10 min at RT, followed by sonication at 4°C using Bioruptor (Diagenode) for 10 min with 30 s on/off cycles at high intensity. This treatment consistently yielded fragments between 80–100 bp. Finally, EdU-labeled DNA was pulled down by incubation with Streptavidin-agarose beads (Millipore) overnight at 4°C. The beads were washed once with lysis buffer, once with 1 M NaCl, and twice with lysis buffer. Bound proteins were eluted in 2 x NuPAGE LDS sample buffer (Invitrogen) containing 1 x sample reducing agent (Invitrogen) at 95°C for 35 min before loading for western analysis. Protease and phosphatase inhibitors (Thermo Scientific) were added to all buffers. Each iPOND assay was performed 3 times.

### End Resection Assays

End resection was analyzed using two methods. First, end resection following gamma-irradiation was assessed using a single strand BrdU assay as described [51]. To detect single strand DNA formation, A549 cells were transfected with control and EEPD1 siRNAs, and plated on coverslips at 24 h, then incubated with 30 μM BrdU for 42 h before treatment with 20 Gy IR. At various times after irradiation, cells with native (non-denatured) DNA were analyzed by immunofluorescent confocal microscopy to detect BrdU in SS DNA created by end resection.

Second, end resection was also measured adjacent to a specific I-SceI-induced DSB by quantitative PCR (qPCR) [50,53]. Genomic DNA (gDNA) was extracted from HT1904 cells [52] harvested 4 h after infection with adenovirus vectors that express I-SceI (Adv-I-SceI) or GFP (Adv-GFP) as control. Half of the gDNA was digested with *Xma*I (NEB), and the remaining half was mock-digested. PCR reactions included *Xma*I-digested or undigested gDNA as template, 0.5 uM of each primer, 0.2 uM TaqMan probe, and 1X TaqMan universal master mix (ABI). qPCR was performed on a 7900HT Fast Real-Time PCR System (ABI) under standard thermal cycling conditions. Results were analyzed with SDS2.3 (ABI) and Graph Pad 6. For each sample, a ΔCT was calculated by subtracting the CT value of the undigested sample from the CT value of the *Xma*I-digested sample. The percentage of SS DNA was calculated with the following equation: SS DNA% = 1/(2^(ΔCt-1)+0.5)*100 [50]. Primers and probes were: forward (CGACCTTCCATGACCGAGTACAA), reverse (TCCGGGTCGACGGTGTG), and probe (6FAMACCGCGACGACGTCCCCCGGGCC-TAMRA). All Ct values were corrected for different DNA concentrations, as determined by qPCR of a ‘No Cut’ amplicon on chromosome 22 that lacks *Xma*I sites: forward (ACATTGTCTCTGTCATGGGC), reverse (TGTGTCAGGGATTTGCTCAC), and probe (6FAM AGAGCATGGGTGGATCCTGGATATTCA-TAMRA). DSB induction by Adenoviral-I-SceI was measured by qPCR and calculated as described [52] using a primer set that flanked the I-SceI site, and primers to the chromosome 22 ‘No Cut’ amplicon as a negative control. The ‘No Cut’ amplicon was used to normalize the amount of genomic DNA in the qPCR reaction, and the percentage of DSBs in Adv-GFP treated cells was set to zero. Both end resection assays were performed three times in triplicate.

### Reversed Replication Fork Nuclease Assays

Pure recombinant human FLAG-tagged EEPD1 protein was generated in 293 cells and purified as we described [84]. Nuclease assays were performed as we described [32,84]. 3’ overhang reversed fork (“chicken foot”) structures were obtained by annealing SHL101, SHL108, SHL109, and SHL110, and then gel-purifying the annealed structure. 5’ overhang reversed fork structures were obtained by annealing SHL101, SHL108, SHL111, and SHL112 and then gel-purifying the intact annealed structure [32,84]:

SHL101 (60mer): 5’-CGATACTGAGCGTCACGGACTCTGCCTCAAGACGGTAGTCAACGTGTTACAGACTTGATG-3’

SHL108 (60mer): 5’-CTAGACTCGAGATGTCAAGCAGTCCTAACTTTGAGGCAGAGTCCGTGACGCTCAGTATCG-3’

SHL109 (60mer): 5’-CATCAAGTCTGTAACACGTTGACTACCGTCGATCCACTAG AGGTCTAAGCGACCTCATTC-3’

SHL110 (40mer): 5’-CTAGTGGATCAGTTAGGACTGCTTGACATCTCGAGTCTAG-3’

SHL111 (40mer): 5’-CATCAAGTCTGTAACACGTTGACTACCGTCGATCCACTAG-3’

SHL112 (60mer): 5’-AGGTCTAAGCGACCTCATTCCTAGTGGATCAGTTAGGACTGCTTGACATCTCGAGTCTAG

### Homologous Recombination (HR), Non-homologous End-Joining (NHEJ), and Micro-homology End Joining (MMEJ) Assays

The HT256 reporter system was used to determine I-SceI-induced HR efficiency and gene conversion tract spectra as we described [39,85]. The EJ5-GFP U2OS system was used to assess NHEJ [37,38]. Both of these reporter systems have single, integrated copies of reporters with I-SceI target sites cleaved upon transfection of an I-SceI expression vector. Cells were transfected with either control or EEPD1 siRNAs or shRNAs, and then transfected 24 or 48 h later with pCBA-SceI or empty vector, using PEI. After 48 h incubation, EJ5 cells were trypsinized and washed with PBS and GFP-positive cells reflecting NHEJ frequencies were measured by FACSort (Becton-Dickinson, San Jose, CA) and analyzed with CellQuest (Becton-Dickinson) software. Productive HR in HT256 cells reconstitutes a functional neomycin phosphotransferase gene, generating G418 resistant colonies. Two thousand cells were plated in three 10-cm dishes per each condition, in non-selective media, 24 h after I-SceI vector transfection to establish plating efficiency. To assess HR, 500,000 cells were plated in media with G418 (325 ug/ml, 100% active) added 24 h after transfection. DSB-induced HR frequencies were calculated as the number of G418-resistant colonies per viable cell plated in G418 medium after 21 days, normalized for plating efficiency. HR assays were performed 15 times in triplicate and the NHEJ assays 12 times in triplicate.

Gene conversion tracts were analyzed as we described [39,40,85] on the above HR repaired neo-positive colonies. HT256 G418 resistant colonies were stained and counted, or expanded under continuous G418 selection for gDNA isolation and molecular analysis. Genomic DNA was extracted using the DNeasy Tissue Kit (Qiagen, Valencia, CA). Primers A (CCTTCACTTTCCAGAGGGTC) and B (GCGAAGAACTCCAGCATGAG) were used to amplify a 1.5 kb fragment comprising the recipient neo allele (MMTVneo) by using standard PCR conditions. The donor neo allele carries 12 silent single-base mutations at approximately 100 bp intervals that create restriction fragment length polymorphisms (RFLPs). These RFLP markers allow high-resolution analysis of gene conversion tract length, directionality, and continuity. The 12 silent RFLP markers and the natural BanII site were mapped in PCR fragments amplified from HR products.

The analysis of MMEJ versus HR competitive repair from a single DSB was performed in modified U2OS cells as we described [19]. To directly compare MMEJ with HR, an EGFP-based MMEJ and HR competition reporter system, termed EGFP-MMEJ/HR-MluI, was generated. This reporter had the EGFP (R-EGFP) cassette of EGFP-HR replaced with the EGFP-MMEJ cassette. A unique MluI site in the parent EGFP (D-EGFP) cassette was created via a silent mutation at the BssHII site. Upon I-SceI cleavage, restoration of a functional EGFP cassette results in loss of the I-SceI site after cells undergo repair by either MMEJ or HR. PCR analysis of the sorted green cells using primers specific for R-EGFP was performed. The primers were: EGFP MMEJ/HR Forward:5’-ACGGGGTCATTAGTTCATAGCCCA, EGFP MMEJ/HR Reverse: 5’-GGGATTTTGCCGATTTCGGCC. Repair of the I-SceI DSB by MMEJ would retain one copy of the 9-bp duplication with an intact BssHII site. The percentage of the BssHII-digestible bands within the total PCR amplified product reflects the MMEJ frequency. Repair of that I-SceI-induced DSB by HR transfers the MluI site from D-EGFP to R-EGFP, and thus the percentage of MluI-digestible bands of the total PCR product reflects the HR frequency.

### Nuclear Structure Analysis

To assess nuclear structural abnormalities, micronuclei and post-mitotic bridging from aberrant chromosomal segregation, control or HU-treated cells, with or without EEPD1 depletion, were grown on coverslips and fixed as above, and stained with 300 nM DAPI (Beckman) in PBS for 5 min [27]. After washing with PBS, coverslips were mounted in anti-fade solution and analyzed within 24 h. Of note, EEPD1 was located in the nucleus, but did not form discrete foci with or without DNA damage. At least ten distinct determinations (142–190 nuclei per determination) were performed for each treatment group.

### Cytogenetic Analysis

Structural aberrations in metaphase chromosomes were scored by Solid Giemsa staining as described [86,87]. EEPD1 and/or 53BP1 were repressed using siRNA in log phase A549 cells, with or without 18 h treatment with 10 mM HU. Cells were washed with PBS and fresh media with colcemid (final concentration 0.25 ug/mL) was added, and cells were incubated for 24 h before harvest. Chromosome preparations were made according to the standard air drying procedure as we described [87]. Cells were harvested, washed with pre-warmed PBS twice, hypotonically treated (0.56% KCl, 20 min at 37°C) and subsequently fixed in freshly prepared acetic acid-methanol (1:3). At least three changes of fixative were performed before the cell suspension was dropped on to a pre-cleaned chilled glass slide and dried at RT at least for 1 day before staining. Structural translocations such as dicentric and ring chromosomes, and Robertsonian translocations, were scored under 63x magnification [87]. Statistics were calculated using Fisher exact tests. Cytogenetic spreads were performed three distinct times with a total of 102–374 metaphase spreads were analyzed per condition.

### Single Cell Electrophoresis (Comet) Assays

Alkaline single cell electrophoresis assays for SS nicking was performed as described [88] using the CometAssay kit (Trevigen, Gaithersburg, MD). Briefly, A549 cells were transfected with siRNAs and treated with 10 mM HU for 1 h or mock treated. Cells were harvested, washed with cold PBS and mixed with molten 1:10 (v/v) LMAgarose and immediately spread over the sample area of comet slides. Cells were immobilized at 4°C in the dark for 30 min and immersed in lysis solution overnight. For the alkaline comet assay, slides were treated with alkaline unwinding solution for 1 h at 4°C in the dark before electrophoresis in alkaline electrophoresis buffer. Samples were rinsed with water and immersed in 70% ethanol before drying at 37°C for 15 min. SYBR Gold was used to stain dried agarose for 30 min at RT before rinsing and drying again. Slides were viewed with a Leica inverted epifluorescence microscope and analyzed by software Image J with OpenComet plugin [89]. Alkaline comet assays were performed five times in triplicate, counting >100 slides per experiment.

### Gene Expression Analysis

Colorectal carcinoma biopsies were re-analyzed specifically for EEPD1 expression, compared to adjacent normal mucosa. Gene expression measurements were performed in 217 patients with colorectal carcinomas from pre-therapeutic biopsies as we described [80]. From 217 patients, tumor samples were extracted, and from 181 of these matched normal tissue (mucosa) samples were also obtained. Gene expression was measured on Agilent Human Microarrays. Microarray data was extracted as log_2_ intensities and quartile normalized. Gene expression of EEPD1 (Agilent Probe: A_23_P333498, Refseq: NM_030636, Chr. Coord: chr7:36340858–36340917, Probe: CAGCCTGTTCTTACTCCAGCTCAACCCATTGGGTGTTGGCTGTTTTTGGTTTTAGTTGTT) was obtained. Significance was computed from matched tumor vs. mucosa samples using a paired Wilcoxon test.

## Supporting Information

S1 Fig EEPD1 domain structure and expression in human tissues and cell lines. **A,** EEPD1 includes two helix-hairpin-helix (HhH) domains related to RuvA2, a conserved D-D-N nuclease active site and a nuclease/phosphatase domain related to DNase I. **B,** Western blots above demonstrating EEPD1 expression in human tissues (left) and human cell lines (right) with beta-actin as loading control. Note: beta-actin is not expressed in heart. EEPD1 mRNA expression in tissues monitored by RT-PCR, with GAPDH as control, is shown below.
(TIF)

S2 Fig EEPD1 depletion slows S-G2/M transition. **A**, Cell cycle profiles of asynchronous A549 cells with or without EEPD1 depletion. **B**, As in panel **A** except cells were synchronized with thymidine and released for indicated times.
(TIF)

S3 Fig EEPD1 is not required for focus formation by early DSB repair components. **A**, BRCA1 and gamma-H2Ax foci in DAPI-stained nuclei were detected by immunofluorescence microscopy in A549 cells treated with 10 mM HU for 18 h with or without EEPD1 depletion. **B**, **C,** as in panel **A** for 53BP1 and NBS1, respectively. For all three data sets, only HU treated cells are shown. Percentages of cells with ≥3 foci shown at right as averages (±SD) for 5–11 determinations (48–127 total cells scored per condition).
(TIF)

S4 Fig EEPD1 is located in the nucleus. **A**, Protein from cytoplasmic (C) or nuclear (N) extracts was analyzed for EEPD1 expression by Western blot in untreated cells (two left lanes) or after 18 h treatment with 1 μM VP-16 and released into fresh growth medium for the indicated times. **B**, GFP-tagged EEPD1 localizes to the nucleus with diffuse expression.
(TIF)

S5 Fig EEPD1 is a nuclease that cleaves 5’ overhangs. Purified recombinant EEPD1 or Metnase nucleases were incubated with 5’ ^32^P-labled (*) reversed replication fork (chicken foot) structures with either 3’ (left) or 5’ (right) overhangs, and reaction products were separated by PAGE. EEPD1 cleaved the 5’ overhang but showed no activity with a 3’ overhang.
(TIF)

S6 Fig EEPD1 depletion with or without co-depletion of CtIP, Dna2, or Exo1 suppresses cell proliferation. EEPD1 was depleted alone or with co-depletion of CtIP, Dna2, or Exo1 using siRNA. Cell proliferation was measured using MTT assays. **A**, EEPD1 depletion and CtIP depletion, alone or together, decrease cell proliferation equally with or without replication stressed induced by treatment with 10 mM HU for 1 h. **B**, Exo1 depletion does not decrease cell proliferation to the same extent as EEPD1 depletion, with or without replication stress. Exo1 is required for proliferation in the face of HU-induced replication stress. **C**, Dna2 is required for proliferation with or without replication stress with HU. Co-depletion of Dna2 and EEPD1 does not further reduce proliferation with or without replication stress. Note that when EEPD1 is depleted, Exo1, BLM and CtIP levels are reduced (panels **A**,**B**, and **Fig 6G**) indicating that expression of these resection factors is coordinately regulated.
(TIF)

S7 Fig EEPD1 depletion with or without co-depletion of CtIP, Dna2, or Exo1 suppresses gamma-H2Ax focus formation in response to replication stress. **A**, Experimental scheme. **B,** Western blots demonstrating single and double knockdown of EEPD1, CtIP, Dna2, and Exo1. **C, D**, Representative gamma-H2Ax results are shown in panel C, and quantitated as percentages of cells with >5 gamma-H2Ax foci/cell are plotted for 6–11 determinations. Statistical significance was calculated by using Mann-Whitney tests.
(TIF)
