# Supplementary figures and images for: EEPD1 Rescues Stressed Replication Forks and Maintains Genome Stability by Promoting End Resection and Homologous Recombination Repair
Source: PLoS Genet. 2015 Dec 18;11(12):e1005675. doi: 10.1371/journal.pgen.1005675 (PMC4684289; doi:10.1371/journal.pgen.1005675)

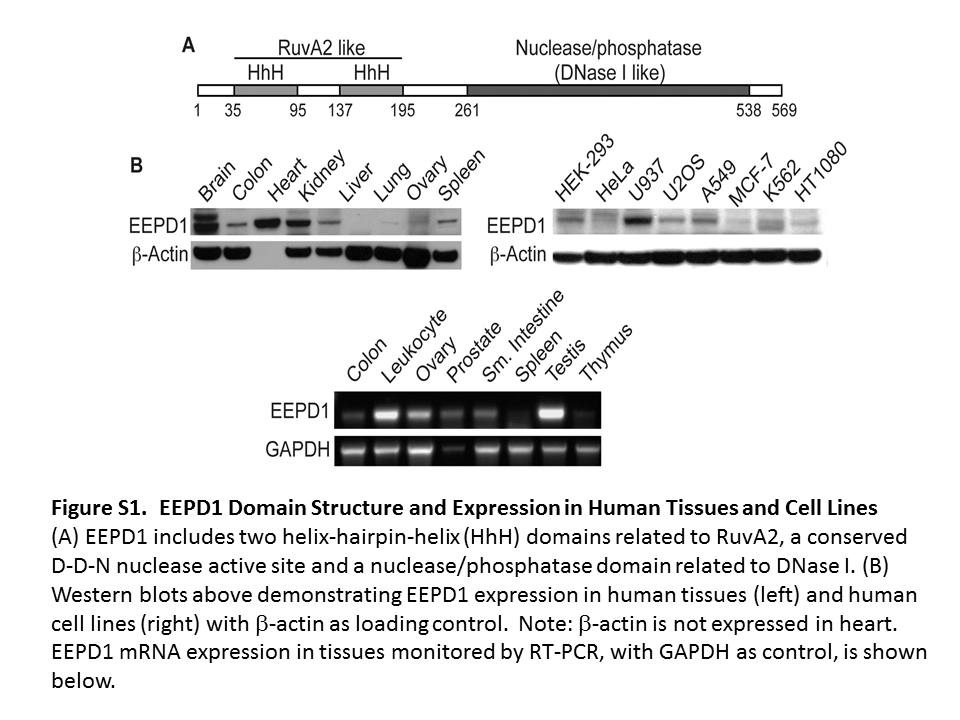

Supplement: S1 Fig — A, EEPD1 includes two helix-hairpin-helix (HhH) domains related to RuvA2, a conserved D-D-N nuclease active site and a nuclease/phosphatase domain related to DNase I. B, Western blots above demonstrating EEPD1 expression in human tissues (left) and human cell lines (right) with beta-actin as loading control. Note: beta-actin is not expressed in heart. EEPD1 mRNA expression in tissues monitored by RT-PCR, with GAPDH as control, is shown below. (TIF) [file pgen.1005675.s001.tif]

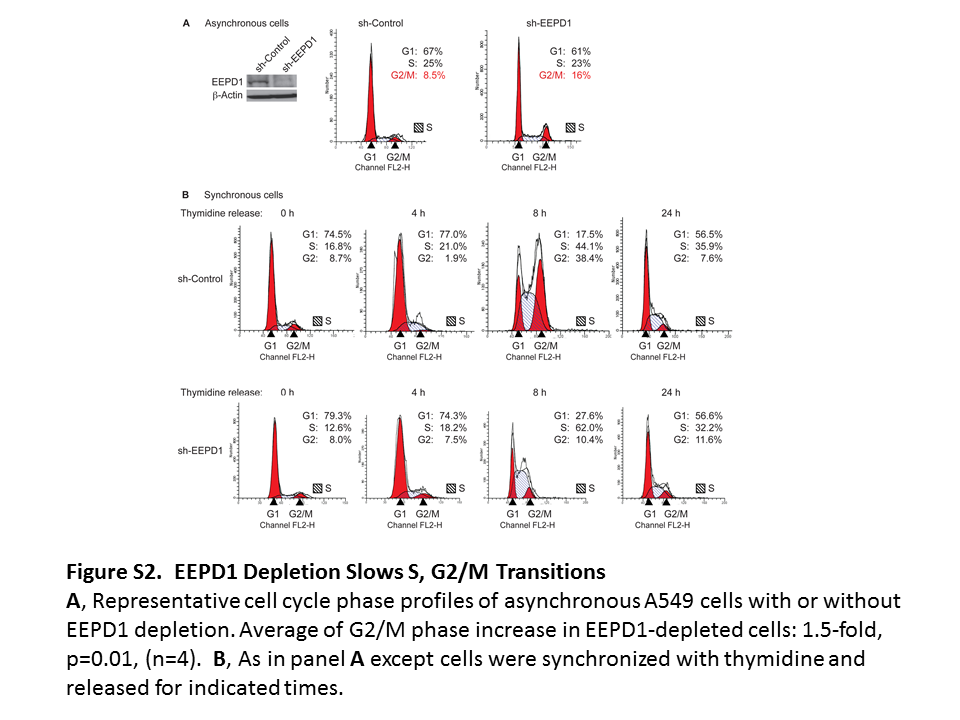

Supplement: S2 Fig — A, Cell cycle profiles of asynchronous A549 cells with or without EEPD1 depletion. B, As in panel A except cells were synchronized with thymidine and released for indicated times. (TIF) [file pgen.1005675.s002.tif]

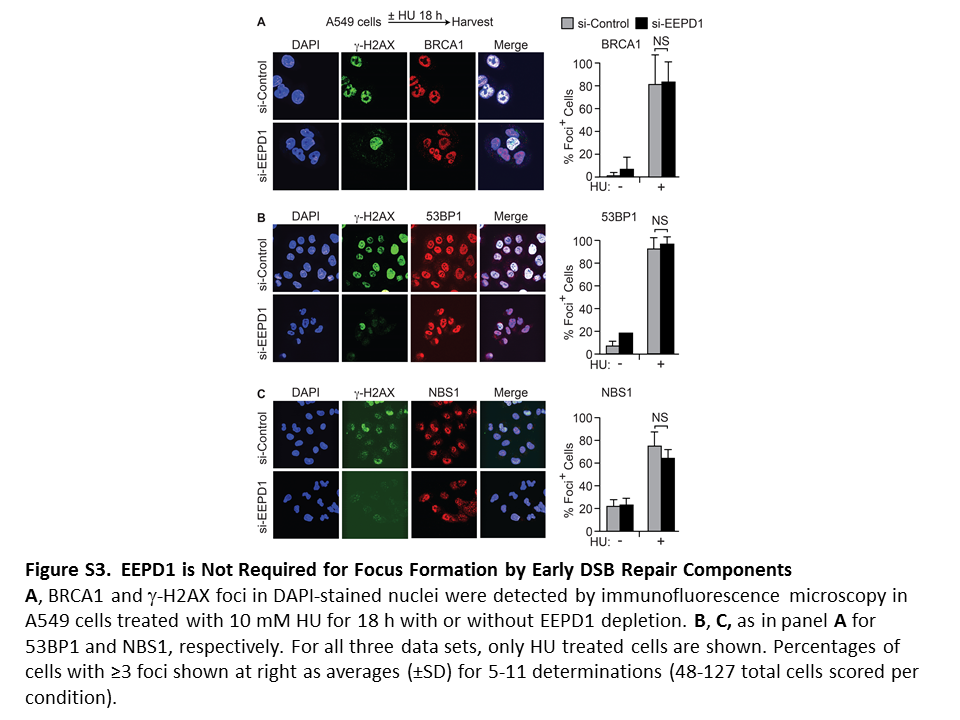

Supplement: S3 Fig — A, BRCA1 and gamma-H2Ax foci in DAPI-stained nuclei were detected by immunofluorescence microscopy in A549 cells treated with 10 mM HU for 18 h with or without EEPD1 depletion. B, C, as in panel A for 53BP1 and NBS1, respectively. For all three data sets, only HU treated cells are shown. Percentages of cells with ≥3 foci shown at right as averages (±SD) for 5–11 determinations (48–127 total cells scored per condition). (TIF) [file pgen.1005675.s003.tif]

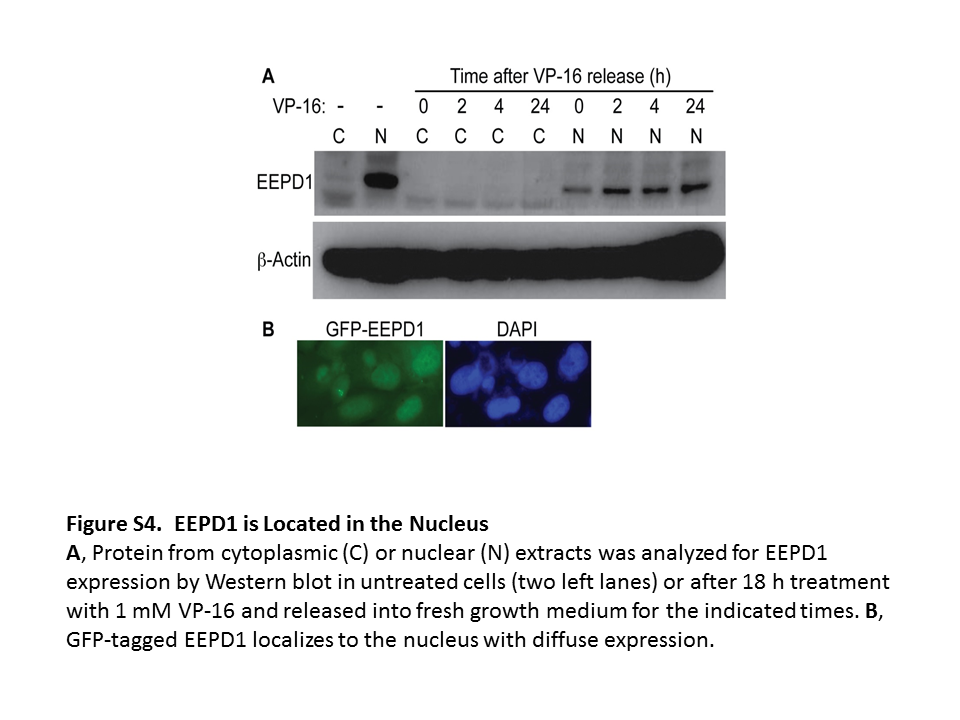

Supplement: S4 Fig — A, Protein from cytoplasmic (C) or nuclear (N) extracts was analyzed for EEPD1 expression by Western blot in untreated cells (two left lanes) or after 18 h treatment with 1 μM VP-16 and released into fresh growth medium for the indicated times. B, GFP-tagged EEPD1 localizes to the nucleus with diffuse expression. (TIF) [file pgen.1005675.s004.tif]

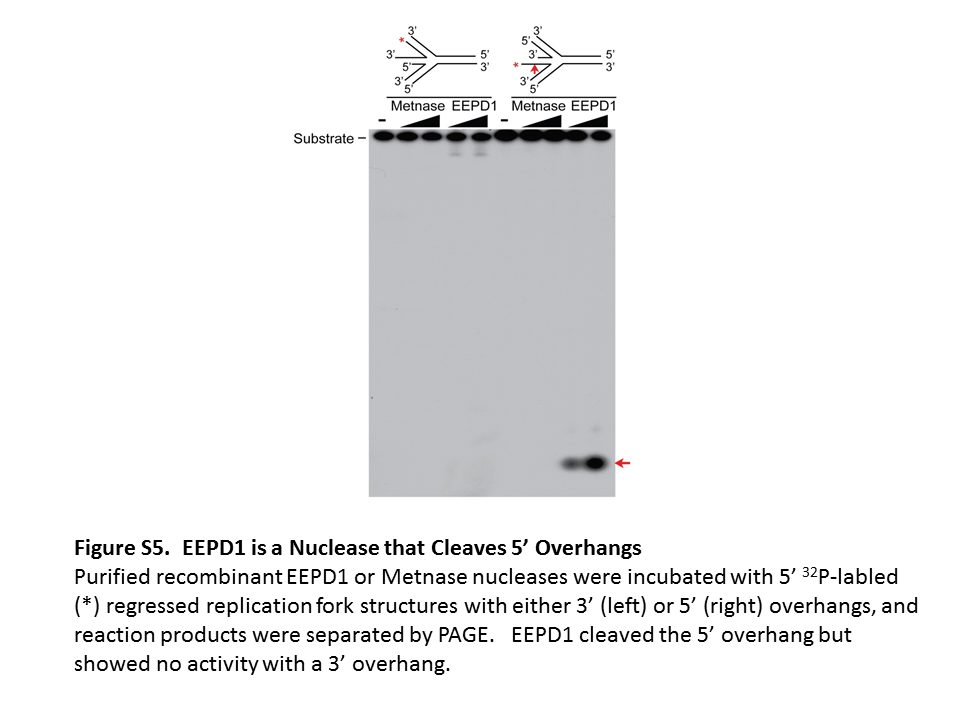

Supplement: S5 Fig — Purified recombinant EEPD1 or Metnase nucleases were incubated with 5’ 32P-labled (*) reversed replication fork (chicken foot) structures with either 3’ (left) or 5’ (right) overhangs, and reaction products were separated by PAGE. EEPD1 cleaved the 5’ overhang but showed no activity with a 3’ overhang. (TIF) [file pgen.1005675.s005.tif]

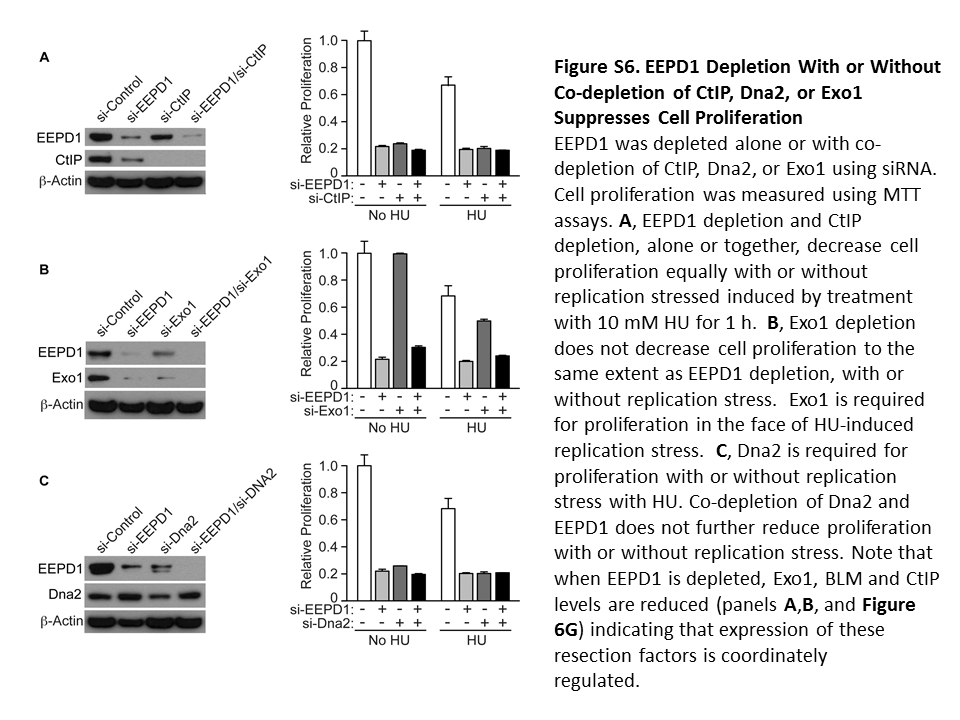

Supplement: S6 Fig — EEPD1 was depleted alone or with co-depletion of CtIP, Dna2, or Exo1 using siRNA. Cell proliferation was measured using MTT assays. A, EEPD1 depletion and CtIP depletion, alone or together, decrease cell proliferation equally with or without replication stressed induced by treatment with 10 mM HU for 1 h. B, Exo1 depletion does not decrease cell proliferation to the same extent as EEPD1 depletion, with or without replication stress. Exo1 is required for proliferation in the face of HU-induced replication stress. C, Dna2 is required for proliferation with or without replication stress with HU. Co-depletion of Dna2 and EEPD1 does not further reduce proliferation with or without replication stress. Note that when EEPD1 is depleted, Exo1, BLM and CtIP levels are reduced (panels A,B, and Fig 6G) indicating that expression of these resection factors is coordinately regulated. (TIF) [file pgen.1005675.s006.tif]

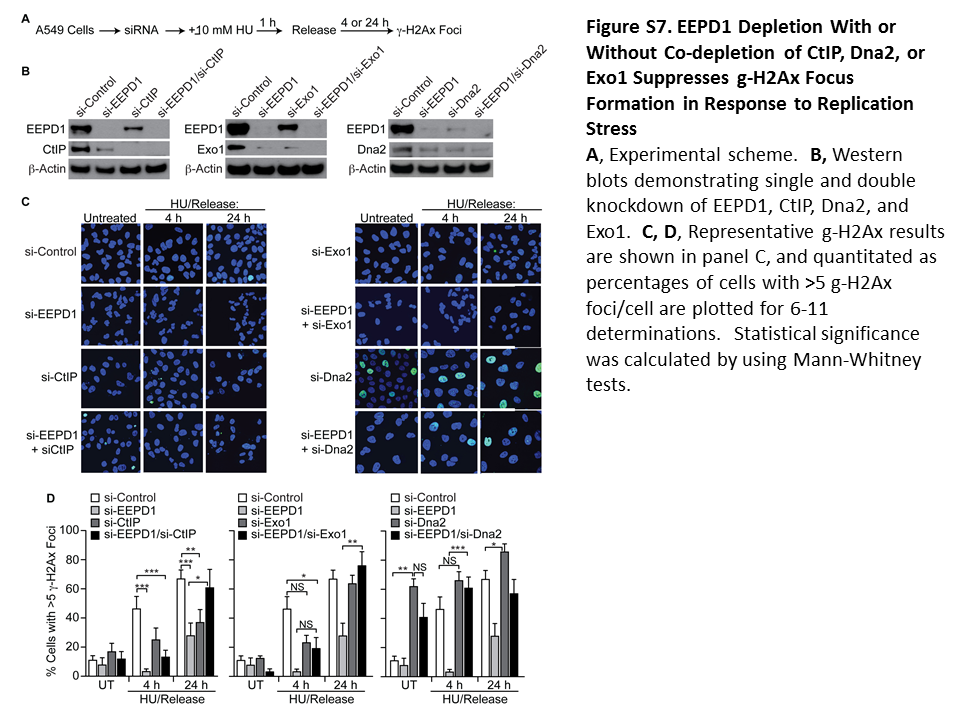

Supplement: S7 Fig — A, Experimental scheme. B, Western blots demonstrating single and double knockdown of EEPD1, CtIP, Dna2, and Exo1. C, D, Representative gamma-H2Ax results are shown in panel C, and quantitated as percentages of cells with >5 gamma-H2Ax foci/cell are plotted for 6–11 determinations. Statistical significance was calculated by using Mann-Whitney tests. (TIF) [file pgen.1005675.s007.tif]
